# Cytoplasmic Restriction of Mutated SOD1 Impairs the DNA Repair Process in Spinal Cord Neurons

**DOI:** 10.3390/cells8121502

**Published:** 2019-11-23

**Authors:** Jiaojie Li, Miyoung Song, Sanghyun Moh, Heemin Kim, Dae-Hwan Kim

**Affiliations:** 1 Department of Chemistry, Gwangju Institute of Science and Technology (GIST), Gwangju 61005, Korea; jjli@gist.ac.kr; 2 Anti-Aging Research Institute of Bio-FD&C Co, Ltd., Incheon 21990, Korea; mysong@biofdnc.com (M.S.); shmoh@biofdnc.com (S.M.); 3 Department of Medicine, Seoul National University, Seoul 03080, Korea; 4 School of Undergraduate Studies, Daegu Gyeongbuk Institute of Science and Technology (DGIST), Daegu 42988, Korea

**Keywords:** amyotrophic lateral sclerosis, superoxide dismutase 1, DNA damage, protein disulphide isomerase, DNA repair

## Abstract

Amyotrophic lateral sclerosis (ALS) caused by mutation of superoxide dismutase 1 (SOD1), affects various cellular processes and results in the death of motor neurons with fatal defects. Currently, several neurological disorders associated with DNA damage are known to directly induce neurodegenerative diseases. In this research, we found that cytoplasmic restriction of SOD1G93A, which inhibited the nucleic translocation of SOD1WT, was directly related to increasing DNA damage in SOD1- mutated ALS disease. Our study showed that nucleic transport of DNA repair- processing proteins, such as p53, APEX1, HDAC1, and ALS- linked FUS were interfered with under increased endoplasmic reticulum (ER) stress in the presence of SOD1G93A. During aging, the unsuccessful recognition and repair process of damaged DNA, due to the mislocalized DNA repair proteins might be closely associated with the enhanced susceptibility of DNA damage in SOD1- mutated neurons. In addition, the co-expression of protein disulphide isomerase (PDI) directly interacting with SOD1 protein in neurons enhances the nucleic transport of cytoplasmic- restricted SOD1G93A. Therefore, our results showed that enhanced DNA damage by SOD1 mutation-induced ALS disease and further suggested that PDI could be a strong candidate molecule to protect neuronal apoptosis by reducing DNA damage in ALS disease.

## 1. Introduction

Amyotrophic lateral sclerosis (ALS) is a well-known motor neurone disease, resulting in a decrease in the number of upper and lower motor neurons. Unlike other neuronal diseases, such as Parkinson’s, Alzheimer’s and Huntington disease, ALS is a relatively rare neurological disease, but it results in the serious loss of vital within five years [1,2]. Nowadays, 1–2 per 100,000 people are diagnosed with ALS with a confirmed diagnosis, usually at a relatively late stage [1,3]. Alongside the common symptoms of the disease, such as rapid loss of muscle power, atrophy and paralysis, respiratory failure, due to dysfunctional respiratory muscles eventually results in the death of the patient. Around 90% ALS occurrence is due to unknown causes and sporadic (sALS), but the remaining 10% of ALS, familial ALS (fALS), is due to genetic disorders, such as gene mutations [4,5]. Several mutations in various genes, including superoxide dismutase 1 (SOD1), fused in sarcoma (FUS) TAR DNA-binding protein 43 kD (TDP-43), etc., are associated with initiation and progression of fALS [6].

Since the first report of SOD1 mutation being related to ALS in 1993, over 150 mutations in SOD1 have been identified in ALS patients. Such SOD1 mutations are found in around 20% of fALS and 3% of sALS cases [7,8]. In the SOD1-mutated mouse model, degeneration of motor neurons is caused by toxic gain-of-function, wherein the mutated SOD1 induces malfunctioning in motor neurons [9]. A study of 40 fALS cases has shown mutations in TDP-43 or FUS and dysfunctional RNA-binding proteins, due to the mutations as being directly associated with ALS induction. In the case of TDP-43, most mutations are enriched in the glycine-rich C-terminal domain, but mutations of FUS are often observed at the nucleic localization signal sequence region. Such mutations influence the protein structure or cellular localization of RNA-binding proteins [10]. The TDP-43 mutation is detected in 3% fALS and 1% sALS cases, whereas, the identified FUS mutation is relatively high in fALS (5%), but rare in sALS (<1%) [11]. In contrast to SOD1 mutation-induced ALS, the loss or reduced function of FUS or TDP-43 causes ALS, due to neuronal death [6].

The nucleic localized TDP-43 and FUS in repairing damaged DNA show the numerous genetic interactions between them. The physical interaction of both RNA binding proteins is required for the expression of histone deacetylase (HDAC) 6 [12,13]. The mutated TDP-43 is also reported to form a binding complex with normal FUS, thereby accelerating the ALS-pathogenic process [14]. In addition, ectopic coexpression of WT FUS with mutated TDP-43 and WT TDP-43 with mutated FUS can both enhance TDP-43 or FUS related ALS disease symptoms, compared with the phenotype of overexpression of mutated FUS or TDP-43 [15]. Thus, single-mutated RNA binding protein, FUS or TDP-43 increases ALS-pathogenicity by cooperating with another normal RNA binding protein. In the genetic interaction analysis between TDP-43 and FUS associated with pathogenic progression of ALS, TDP-43 functions as an upstream molecule for FUS [16].

Unlike nucleic localization of RNA binding proteins, TDP43 and FUS, ubiquitously expressed SOD1 is present throughout neurons, including in the organelles, such as the nucleus, lysosome, intermembrane space of the mitochondria, and the cytoplasm [17]. The evidence of genetic association of SOD1 with RNA binding proteins have been recently reported. The mutation of FUS or TDP-43 limits the splicing variety of SOD1 RNA [18]. Moreover, SOD1 mRNA level is controlled by the quantity of TDP-43 protein; for example, the knocked-down TDP-43 increases SOD1 mRNA in a cultured cell model [19]. In addition, mutual physical interactions, the mutated SOD1 which misfolds structure of normal RNA-binding proteins, and the mutated RNA-binding proteins which misfold structure of normal SOD1, have been reported related to motor neuron pathology in ALS [20,21].

Currently, DNA damage in ALS is controversial. In contrast to previous studies, DNA damage was not investigated in the SOD1G93A fALS mouse model [22]. Nonetheless, other studies examined the increased susceptibility to DNA damage in ALS. In this study, we showed that cytoplasmic localization of mutated SOD1 inhibited the nucleic localization of WT SOD1, subsequently induced ER stress given the increased expression of cytoplasmic SOD1 protein, and thus, resulted in eventual DNA damage. Unlike TDP-43, one of the ALS-related DNA repair proteins, the restricted cytoplasmic localization of FUS and APEX1 by SOD1 mutation is likely to accelerate DNA damage. However, overexpression of PDI, which interacts directly with SOD1 protein rescues the cytoplasmic mislocalization of SOD1G93A. Our findings identify that DNA damage is related to the mislocalization of mutated SOD1 and the dysfunctional interactive correlation between mutated SOD1 and DNA repair proteins contribute to DNA damage. PDI, acts as a strong rescue candidate molecule to translocate the mutated SOD1 protein into the nucleus.

## 2. Materials and Methods

### 2.1. Embryo Genotyping

Genomic DNA was isolated from tissue dissected from fetal mouse, using the DNeasy Blood and Tissue Kit (Qiagen, Hilden, Germany) and purified after overnight incubation of the tissue at 55 °C. A PCR mixture with 1 μL purified genomic DNA was used as the template, and two pairs of primers were used to check the genotype of SOD1 transgenic (TG) mouse. The primer sequences are, as follows: 5′-CATCAGCCCTAATCCATCTGA-3′ as forward and 5′-CGCGACTAACAATCAAACTG-3′ as reverse for hSOD1, and 5′-CTAGGCCACAGAATTGAAAGATCT-3′ as forward and 5′-GTAGGTGGAAATTCTAGCATCATCC-3′ as reverse for mSOD1.

### 2.2. Primary Culture of Neurons

The care guideline of experimental animals was approved by the Animal Care Committee of the Seoul National University. Pregnant B6SJL-TG (SOD1)/B6SJL mice were humanely killed according to the guidelines of the Institute Animal Care and Use Committee (IACUC, #001169, 2010). Primary cultured neurons were prepared from 16-day-old fetal mice. A modified protocol was used for primary neuron culture. We removed the skin from the dissected fetus head and isolated the fetal brain by squeezing the head in Ca^2+^/Mg^2+^-free Hanks Balanced Salts (Gibco, Grand Island, NY, USA). After removing the meninges, the brain tissue was incubated with 2 mL enzyme solution, including 20 U Papain, 1 μM CaCl_2_, and 1 μM EDTA in 2 mL HBSS per one fetal brain tissue at 37 °C for 15 min. After discarding the enzyme solution, 2 mL diluted trypsin inhibitor (Sigma, Saint Louis, MI, USA) with neurobasal media (Gibco, Grand Island, NY, USA); two-fifths of the above solution was added and incubated at 37 °C for 15 min. Then, the brain tissue was dissociated into single cells by triturating 10–15 times with 1000 and 200 P tip. The neuronal cell pellet was resuspended in neuronal growth media: Neurobasal media (Gibco) and B27 (Gibco) after centrifuging at 1000 rpm for 5 min for cell collection. After cell counting, 5 × 10^6^ cells were seeded onto a coverslip coated with poly-d-lysine (Sigma) in 6-well plates. One-half of the neuronal growth media was changed every 2–3 days to maintain the primary cultured neurons.

### 2.3. NSC34 Cell Culture

NSC34 cells were cultured in DMEM media, including 10% FBS, 1% penicillin–streptomycin and 1% NEAA (Thermo-fisher, Wilmington, MA, USA). The cells were initially seeded at 10% confluency in 10 cm cell culture dishes and passaged every four days.

### 2.4. Plasmid Constructions

All human genes used in our experiments were purchased from Addgene. Full length SOD1WT and SOD1G93A (missing a stop codon) were inserted into EcoRI and HindIII of pEGFP-N1 (Clontech, Palo Alto, CA, USA) to generate GFP-tagged hSOD1WT and SOD1G93A. SOD1WT or SOD1G93A-RFP was generated by replacing the GFP with RFP by cutting with BamHI and NotI. hTDP-43, hFUS, hHDAC1, hOGG1, hPARP1, and hXRCC1 without a stop codon were cloned into pEGFP-N1 with XbaI and SalI or HindIII. SOD1WT-RFP, including the internal ribosome entry site (IRES) sequence, was inserted into the cloned hSOD1G93A-GFP with NheI and XhoI. mCherry tagged TDP-43, FUS, and PDI were inserted into IRES-SOD1G93A-GFP by replacing SOD1WT-RFP using SacII and BamHI.

### 2.5. Transfection of Plasmids

All the manipulated plasmids were transfected in the primary cultured neurons and NSC34 cells by using the lipofectamin 2000 reagent (Invitrogen, Carlsbad, CA, USA). Five μg plasmids were mixed with 50 μL of Opti-MEM I medium and 10 μL lipofectamine 2000 was mixed with 50 μL of Opti-MEM I medium. After incubation for 5 min, we combined the plasmid and lipofectamine 2000. The resulting mixture was further incubated for 20 min at room temperature and then transferred into each well containing cells and medium. After incubation in a CO_2_ incubator at 37 °C for 12 h, fresh medium was added. The FACS analysis was used to check the transfection efficiency (Figure S1). We fixed both neurons and cells with 4% paraformaldehyde in PBS for 10 min and used both confocal and multi-photon microscopy, MRC-1024 LaserSharp 2000 (Bio-Rad, Hercules, CA, USA) to check protein fluorescence.

### 2.6. Immunostaining

Immunostaining was performed using the following antibodies: hSOD1 (1:50, Cell Signaling, Danvers, MA, USA); PDI (1:100, ENZO, Farmingdale, NY, USA); ATM (1:100, Abcam, Cambridge, MA, USA); phosphorylated histone protein (pH2A.X) (1:250, Cell signaling, Danvers, MA, USA); and Map2 (1:500, Thermo Scientific, Waltham, MA, USA). Primary cultured neurons were fixed with 4% paraformaldehyde in PBS for 15 min. Blocking was performed using 10% bovine serum albumin (BSA) in PBS-T (0.1% Triton X-100, Sigma, Saint Louis, MI, USA) for 2 h at room temperature. Each primary antibody mixed in blocking solution was incubated with the sample for 2 h and rinsed with PBS-T three times for 15 min. Then, 4’,6-diamidino-2-phenylindole (DAPI) (Sigma, St. Louis, MI, USA) was used for nuclear staining and secondary antibody—Alexa Fluor™ 488 goat anti-rabbit or mouse (1:1000) or Alexa Fluor™ 568 goat anti-mouse or rabbit (1:1000) (Thermo Scientific, Waltham, MA, USA) was used to detect the primary antibody. Confocal and multi-photon microscopy, MRC-1024 LaserSharp 2000 (Bio-Rad, Hercules, CA, USA) was used to obtain the staining images.

### 2.7. Measurement of Intracellular Ca^2+^ Concentration

SOD1WT-RFP or SOD1G93A-RFP was transfected into the primary cultured neurons and incubated overnight. The fluo-4 direct calcium assay kit (Invitrogen) was used to check the intracellular Ca^2+^ concentration in neurons, as per the following protocol: Two equal volumes of 500 μL 2 X fluo-4 direct™ calcium reagent loading solutions were added into the neuron culture medium, and the plates were incubated at 37 °C for 60 min. Ca^2+^ images was obtained with Eclipse Ti-U (Nikon, Melville, NY, USA).

### 2.8. Immunohistochemistry

Briefly, 4% paraformaldehyde diluted in PBS was perfused to fix the mouse spinal cord; the isolated spinal cord was stored in 2% paraformaldehyde at 4 °C overnight. After that, the paraformaldehyde solution was changed to 10% sucrose solution, and the tissues were stored at 4 °C for one day, changed to 20% sucrose solution for another day, and finally changed to 30% sucrose solution for the next day. The fixed spinal cord tissue was embedded in OCT compound (Cellpath, Newtown, Powys, UK) in dry ice until completely frozen. The prepared sample was stored at −80 °C before sectioning with a microtome. Tissues were sectioned to be 12-μm-thick with the cryostat microtome (Leica, Wetzlar, Germany) and placed on the glass slide. We blocked the dried tissue samples with the blocking reagent in TBS buffer (50 mM Tris-Cl, pH 7.5. 150 mM NaCl) that included 10% BSA and 0.3% Triton for 10 h. The tissues were then incubated with primary antibodies overnight at room temperature. The following primary antibodies were used: p53 (1:250, Abcam, Cambridge, MA, USA); ATM (1:100, Abcam); pH2A.X (1:250); NeuN (1:200, Abcam); TDP43 (1:100, Abcam); FUS (1:100, Novus, Centennial, CO, USA); and ChAT (1:50, Invitrogen). Alexa Fluor™ 488 goat anti-rabbit or mouse (1:1000) and Alexa Fluor™ 568 goat anti-mouse or rabbit (1:250) (Thermo Scientific), and DAP1 were used to detect the primary antibodies and stain nuclei, respectively, for 2 h at room temperature. Confocal and multi-photon microscopy, MRC-1024 LaserSharp 2000 (Bio-Rad, Hercules, CA, USA) was used to examine the stained images. The p-H2Ax positive cells were measured in the ventral horn region in the spinal cord of WT and SOD1G93A transgenic mice.

### 2.9. Western Blotting

Mouse spinal cord tissues was homogenized with lysis buffer (20 mM Tris, pH 8.0; 150 mM NaCl; 0.5% Nonidet P-40; 0.5% sodium deoxycholate) and the proteins were extracted after removing cell debris. The total protein was quantified with a bicinchoninic acid protein assay kit (Pierce, Rockford, IL, USA), and then, 30 μg of protein was subjected to SDS-PAGE. After electrophoresis, the separated protein was transferred onto nitrocellulose membranes. After the membrane was blocked for 1 h in Tris-buffered saline (TBS) containing 0.1% Tween and 10% nonfat dried skim milk, it was incubated with each primary antibody for 2 h at room temperature. The primary antibodies used in western blotting analysis p53 (1:500, Abcam); pH2A.X (1:500, Cell signaling); and PUMA (1:200, Abcam). After washing with TBS-T three times for 15 min, the membrane was incubated for 1 h with the appropriate amount of horseradish peroxidase-conjugated secondary antibody. Anti-βII-tubulin antibody (1:4000) (Santa Cruz, Santa Cruz, CA, USA) was used to detect tubulin protein as the protein loading control, and the chemiluminescence system (GE Healthcare, Little Chalfont, Buckinghamshire, UK) was used to obtain protein bands.

### 2.10. Yeast Two-Hybrid Assay

Yeast two-hybrid analysis was used to examine protein-protein interaction. The full length of SOD1WT and SOD1G93A were cloned into pGBKT7, and the full length PDI was cloned into pGADT7 (Clontech, Palo Alto, CA). Yeast Y2HGold strain was used for transformation of the manipulated plasmids with lithium acetate method according to the manufacturer’s instructions (Clontech, Palo Alto, CA). The co-transformed yeast cells were selected by growth on defined media lacking leucine and tryptophan. Each interaction between SOD1WT or SOD1G93A with PDI protein was monitored by growth on the media with X-alpha-Gal (Takara Bio, Mountain View, CA, USA) but without leucine, tryptophan, and histidine.

### 2.11. FACS Analysis

To statistically analyze DNA damage in neurons, we counted the number of pH2A.X positive neurons after treating neurons with the hydrogen peroxide solution and thapsigargin (both, Sigma). The neurons were isolated and fixed individually with 4% paraformaldehyde for 10 min. After staining neurons with pH2A.X and the secondary antibody, we measured the number of positive neurons with BD FACSCalibur™ (BD Biosciences, San Jose, CA, USA). To measure cell apoptosis, annexin V-FITC (BD Biosciences) was used to stain the isolated neurons, without the fixation step, and BD FACSCalibur™ was used to count the number of apoptotic neurons. SOD1G93A-expressing NSC34 cells and SOD1G93A-GFP- and PDI-mCherry-co-expressing cells were sorted out with BD FACS Aria™ III (BD Biosciences). The sorted cells were amplified and treated with thapsigargin to check for cell apoptosis. After isolating individual cells, Alexa fluor^®^ 647 conjugated annexin V was used to label the apoptotic cells without the fixation step, and BD FACSCalibur™ was used for cell counting.

### 2.12. Statistical Analysis

The *t*-test was used for data analysis. All values are represented as the mean ± standard deviation (SD) in multiple trials, and a *p*-value < 0.05 was considered to indicate statistical significance.

## 3. Results

### 3.1. Presence of SOD1G93A Inhibits Nucleic Localization of SOD1WT

**Figure 1:**
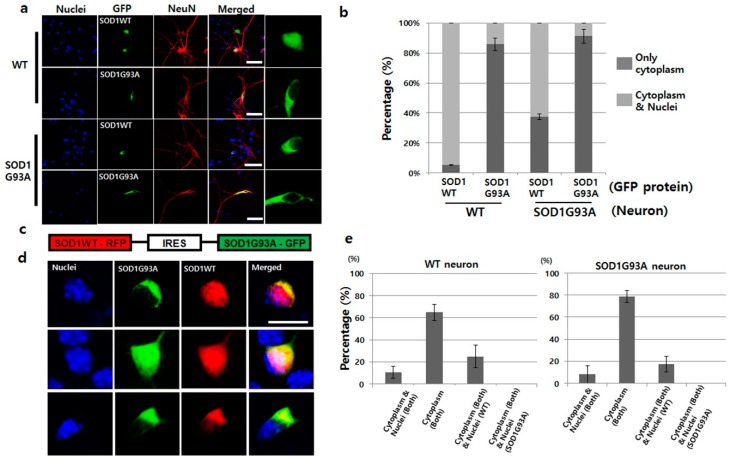
SOD1G93A interfered with the nucleic localization of SOD1WT. (**a**) SOD1WT-GFP was normally localized in the nuclei of WT primary cultured neurons (first layer panel), whereas, SOD1G93A-GFP failed to enter the nuclei of same neurons (second layer panel). SOD1WT-GFP failed to enter the nuclei of SOD1G93A genotype neurons (third layer panel), and most of the SOD1G93A-GFP was localized in the cytoplasm of SOD1G93A genotype neurons (fourth layer panel). (scale bar is 50 μm) (**b**) Statistical analysis on the localization of SOD1WT and G93A-GFP in primary cultured WT and SOD1G93A genotype neurons (three time trials). Dark gray: Localization of cytoplasm and nuclei; weak gray: Nucleic localization only. (*n* = 150 for SOD1WT-GFP expressing WT neurons, 143 for SOD1G93A-GFP expressing WT neurons, 165 for SOD1WT-GFP expressing SOD1G93A background neurons and 159 for SOD1G93A-GFP expressing SOD1G93A background neurons, error bars: Standard deviation). (**c**) Plasmid constructed for expression of SOD1WT-RFP and SOD1G93A-GFP. IRES was used for co-expression to connect the two genes, SOD1WT-RFP and SOD1G93A-GFP. (**d**) Three different localization patterns of SOD1 WT-RFP (red) and SOD1G93A-GFP (green) co-expressed in primary cultured neurons. SOD1G93A-GFP was localized in the cytoplasm, whereas, SOD1WT-RFP was detected in the whole cell (upper). In a few cases, SOD1WT-RFP and SOD1G93A-GFP were colocalized in the whole area, but in most cases, cytoplasmic colocalization of SOD1 WT-RFP and SOD1G93A-GFP was detected (down). (scale bar is 10 μm). (**e**) Statistical analysis on the localization of SOD1WT-RFP and SOD1G93A-GFP in primary cultured neurons (results in triplicates); Left: WT neurons; Right: SOD1G93A genotype neurons. (*n* = 157 for WT neurons and 175 for SOD1G93A background neurons, error bars: Standard deviation).

As previously reported in the cell culture and animal model, mutated SOD1 is mainly located in the cytoplasm, whereas, SOD1WT is present in the whole cellular area [9]. To confirm the locations of both wild type and mutated SOD1 in cells, we tested the localization of the prepared GFP-tagged SOD1WT and SOD1G93A in the primary cultured WT neurons. SOD1WT-GFP was detected in both the nucleus and cytoplasm, but the localization of SOD1G93A-GFP was restricted only to the cytoplasm (Figure 1a). Clearly, the restricted cytosolic localization of SOD1G93A commonly occurs. Based on statistical analysis, 40% of SOD1WT-GFP was found to localize only in the cytoplasm in expression tests with SOD1G93A genotype neurons. The mislocalization ratio was eight-times higher than that in WT neurons (Figure 1b). However, over 85% of SOD1G93A-GFP was localized only in the cytoplasm in primary cultured WT and SOD1G93A genotype neurons (Figure 1a,b). Unlike WT neuron, the restricted localization of SOD1G93A in cytoplasm inhibited the nuclear transportation of SOD1WT. With the specific antibody capable of recognizing the C-terminal domain of normal SOD1 protein, previous studies suggested that the mutated SOD1 misfolded the normal SOD1 [23]. Thus, the large percentage of cytoplasmic localization of SOD1WT in the SOD1G93A genotype neurons indicate that the misfolded SOD1WT by SOD1G93A could be associated with mislocalization of SOD1WT and eventually, cytoplasmic location of SOD1G93A interfere with the nuclear transportation of SOD1WT (Figure 1a). Moreover, most SOD1G93A proteins recognized by the hSOD1-specific antibody were localized in the cytoplasm in SOD1G93A genotype neurons cultured from the SOD1G93A TG fetus (Figure S2a). In the localization test with NSC34 cells, wherein hybrid cell line was fused with neuroblastoma N18TG2 and motor neurons of the mouse spinal cord [24], the localization of SOD1WT-GFP was observed in the whole area in NSC34 cells, while the localization of SOD1G93A-GFP was restricted to the cytoplasm (Figure S2b). These results confirm that the restricted cytoplasmic localization of SOD1G93A, the conformational changed SOD1, is a general phenomenon, but not a cell-type-specific result.

It is well known that the toxic gain-of-function by one copy SOD1 mutation in which the protein level is maintained equal between SOD1WT and mutated SOD1 in a single neuron, induces ALS [25]. However, artificially induced fALS animal disease model consists of the enriched SOD1G93A owing to the overexpression of SOD1G93A, and thus, contains unequal protein concentrations of SOD1WT and SOD1G93A. Therefore, previous results did not accurately reflect the actual disease initiation and progression in the SOD1G93A-induced fALS. To address this limitation, we manipulated the plasmid, wherein SOD1WT and SOD1G93A were connected with an IRES, thereby resulting in equal expression of SOD1WT and SOD1G93A proteins by the single CAG promoter in a single neuron (Figure 1c). Indeed, GFP- and RFP-tagged proteins were co-expressed in the transiently transfected single neuron with the manipulated plasmid (Figure 1d). In the measurement of the RNA level of GFP and RFP region of plasmid by the RT-qPCR, the expression level was almost the same (Figure S3). The localization patterns of SOD1G93A-GFP and SOD1WT-RFP in WT neurons were divided into three types: First, 10% neurons showed localization of SOD1WT and SOD1G93A in the whole neuron; second, 23% neurons demonstrated cytoplasmic localization of SOD1G93A and the presence of SOD1WT in whole neurons; third, 65% neurons, the largest fraction, displayed colocalization of both SOD1G93A and SOD1WT in the cytoplasm alone (Figure 1d,e). In the SOD1G93A genotype neurons, translocation of SOD1WT-RFP into nuclei was still more reduced, and thus, cytoplasmic localization was increased (Figure 1e). In addition, 93% of SOD1G93A genotype neurons demonstrated cytoplasmic localization of SOD1G93A-GFP in single gene expression plasmid, which decreased to 80% if co-expressed with SOD1WT-RFP (Figure 1b,e). Such reductions in the cytoplasmic localization of SOD1G93A-GFP by co-expression of SOD1WT-RFP occurred in WT neurons as well (Figure 1b,e). Thus, cytoplasmic segregation of SOD1WT under enhanced SOD1G93A protein levels become worse, but somehow, increased SOD1WT reduced the cytoplasmic localization of SOD1G93A. Interestingly, in the fourth case, only SOD1WT-RFP was restricted to the cytoplasm, whereas, SOD1G93A was present in the whole neuron; this was not observed in either genotype of neurons (Figure 1e).

### 3.2. Presence of SOD1G93A Sequesters the Upregulated p53 Responding to DNA Damage in the Cytoplasm

**Figure 2:**
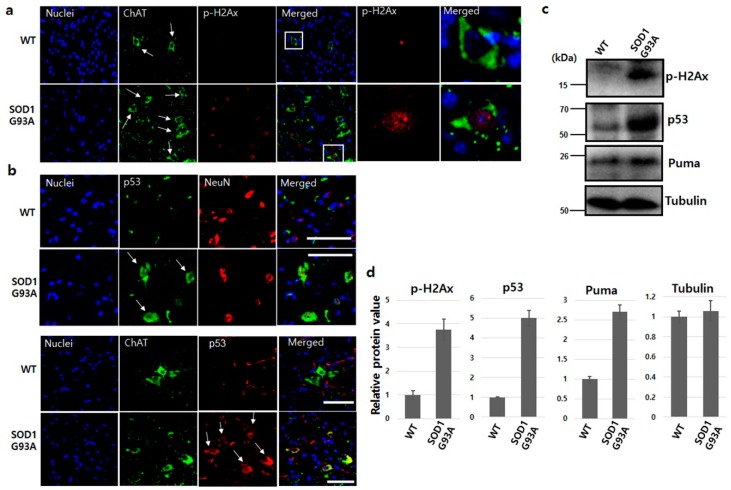
SOD1G93A enhanced DNA damage in the spinal cord. (**a**) Increased DNA damage in motor neurons of SOD1G93A transgenic mouse. The p-H2Ax (red) was examined in motor neurons (green, white arrow) of spinal cord dissected from WT (upper) and SOD1G93A transgenic mice (down) aged 70 days. The significantly enriched p-H2Ax (white square) was observed in motor neurons of the spinal cord dissected from SOD1G93A transgenic mice aged 70 days. (scale bar is 100 μm). (**b**) Increased cytoplasmic p53 protein in SOD1G93A genotype neurons (upper panel, red) and motor neurons (down panel, green). The upregulated p53 (white arrows, green) was detected in the cytoplasm of neurons expressing SOD1G93A (upper panel, down) unlike WT neurons (upper panel, upper). P53 protein (white arrows, red) increased in SOD1G93A genotype motor neurons (green) (down panel, lower), but was not detected in normal motor neurons (down panel, upper). (scale bar is 100 μm). (**c**) The protein level increased in response to DNA damage. The enhanced p-H2aX, p53, and Puma proteins were detected in spinal cord of SOD1G93A genotype mice on western blotting. (**d**) Densitometric quantification from three independent western blot testes. (error bars: Standard deviation).

Mutated SOD1 generates oxidative stress, forms aggregates, induces excitotoxicity and inflammation, and results in motor neuron death in fALS [26]. In SOD1-mutated fALS animal model and ALS patient’s CSF, the occurrence of the malfunction of the mutated SOD1 is a causative source of DNA damage [27,28]. In DNA double-strand breaks, the ataxia telangiectasia mutated (ATM) kinase recognizes DNA breakage, and the kinase activity of ATM then phosphorylates histone H2Ax, a downstream signal molecule [29]. To evaluate DNA damage, we checked ATM and p-H2Ax in the spinal cord dissected from SOD1G93A TG mice at 70 days of age. Both ATM and p-H2Ax showed high expression in many neurons in the spinal cord of SOD1G93A TG mice, compared with the WT spinal cord (Figure S4a and Figure 2a). Particularly, both ATM and p-H2Ax were strongly detected in the nuclei of ChAT, motor neuronal marker positive cells in the spinal cord of SOD1G93A TG mice (Figure S4b and Figure 2a), indicating that the SOD1 mutation significantly induced DNA damage in neurons.

p53 phosphorylated by ATM is upregulated under various DNA damage stresses and occasionally induces cell apoptosis [30,31,32]. The enhanced p53 protein was detected in both neurons and motor neurons of the spinal cord isolated from SOD1G93A TG mice (Figure 2b). These findings demonstrated that DNA damage enhanced by SOD1 mutation upregulates p53 protein level in both neurons and motor neurons, and also confirms that the increase of p53 protein level is in response to DNA damage stress in the human neuroblastoma cell line [33]. Interestingly, the upregulated p53 protein was localized in the cytoplasm, failing to enter the nucleus in SOD1G93A genotype neurons (Figure 2b). Thus, the sequestered p53 protein in the cytoplasm is in a similar situation as functional inactivation, despite upregulation under DNA damage stress [34]. Next, we also examined DNA damage responsive proteins. The protein levels of p-H2Ax, p53, and PUMA were all upregulated in the spinal cord isolated from SOD1G93A TG mice (Figure 2c). Each protein level of p-H2Ax, p53 and puma in the spinal cord of SOD1G93A genotype mice was approximately 3.8, 5.1 and 2.7 fold higher, respectively, compared to the spinal cord of WT mice (Figure 2d). Therefore, we believe that the sequestered localization of both normal and mutated SOD1 in the cytoplasm by SOD1 mutation is the main reason for upregulated p53 protein, indicating that DNA damage and the localization of upregulated p53 is also restricted to the cytoplasm.

### 3.3. DNA Damage in SOD1G93A Genotype Neurons is Associated with Enhanced Endoplasmic Reticulum (ER) Stress

ALS is generally an age-dependent neuronal degenerative disease, with symptoms clearly appearing in those aged ≥ 50 years, despite carrying the mutated genes since their birth [6]. Thus, no defective DNA was detected by ATM and p-H2Ax antibodies in the primary cultured SOD1G93A genotype neurons (data not shown). Therefore, we needed to induce artificial stress conditions to study the role of SOD1G93A in DNA damage. In NSC34 cells, SOD1 mutation generally reduces the antioxidant activity of SOD1 in nuclei by decreasing the amount of SOD1 protein in the nucleus and thus, is more susceptible to the high concentration of hydrogen peroxide [9]. However, under oxidative stress generated 0.6 mM hydrogen peroxide treatment for three days, there was neither any significant difference in the amount of damaged DNA between WT and SOD1G93A genotype primary cultured neurons nor any difference in cell viability (Figures S5 and S6). This result demonstrates that oxidative stress plays a less effective role in DNA damage and neuronal apoptosis of SOD1G93A mutated neurons [33].

**Figure 3:**
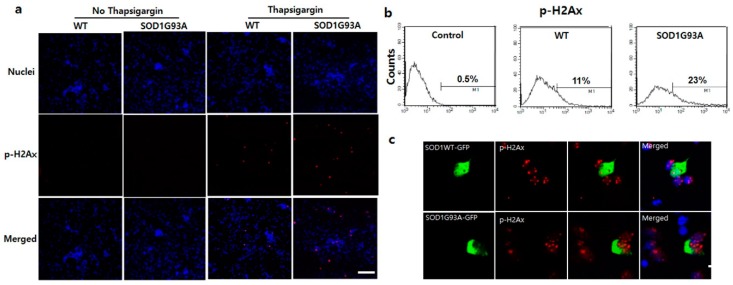
Enhanced susceptibility of DNA damage in SOD1G93A genotype neurons under stress. (**a**) Inducement of DNA damage at 1 μM thapsigargin for 12 h. The p-H2Ax significantly increased in SOD1G93A genotype neurons (fourth column), compared with WT (third column). However, the p-H2Ax was not detected in controls, WT (first column), and SOD1G93A genotype neurons (second column), untreated with thapsigargin. (scale bar is 200 μm). (**b**) FACS analysis to count cell number for DNA damaged neurons after treatment with 1 μM thapsigargin for 12 h. (*n* = 10,000 neurons for WT and SOD1G93A background neurons). (**c**) The nucleic localized SOD1 protein reduces DNA damage. The p-H2Ax staining spots were decreased by the nucleic localization of SOD1WT-GFP (upper), compared with SOD1G93A-GFP (lower). (scale bar is 20 μm).

One of the major cytotoxic effects of SOD1 mutation is ER stress in the motor neurons of fALS [35], and as a result, p53 is upregulated [36]. The cytosolic upregulation of p53 in the spinal cord of SOD1G93A TG mice indicates that the ER stress elevates as the cytosolic SOD1 proteins accumulate in the presence of SOD1G93A (Figure 2b,c). As reported previously, ER stress is directly associated with the elevation of DNA damage, resulting in eventual cell death [37]. To explore the correlation between ER stress and DNA damage in SOD1G93A genotype neurons, we examined DNA damage after treatment of primary cultured neurons with thapsigargin, an ER stress inducer. After treating with 1 μM thapsigargin for 12 h, p-H2Ax protein significantly increased in the primary cultured WT and G93A neurons (Figure 3a). p-H2Ax positive signal was detected in ~23% SOD1G93A genotype neurons, but only in 11% WT neurons (Figure 3b). Additionally, severe breakdown of DNA strands with 1 μM thapsigargin treatment for three days was examined in the SOD1G93A neuron, comparing with WT (Figure S5). Neuronal death was more effectively induced in SOD1G93A genotype neurons than WT after three-day treatments with different concentrations of thapsigargin (Figure S7). These results elucidate that neuronal death and DNA damage in SOD1 mutation-linked ALS are more susceptible to ER stress than oxidative stress, and SOD1G93A genotype neurons are more vulnerable to ER stress than WT neurons.

The level of PDI, one of the strongest indicators for ER stress, is elevated in neurons expressing SOD1G93A [38]. To fully understand the role of SOD1G93A related to ER stress induction, we incubated WT neurons expressing SOD1WT-RFP or SOD1G93A-RFP with 1 μM thapsigargin for 12 h. Under weak ER stress condition, significantly expressed PDI was detected in SOD1G93A-RFP expressing WT neurons, while it was not observed at all in neurons expressing SOD1WT-RFP (Figure S8a). Besides PDI expression, under weak ER stress condition, cytosolic Ca^2+^ levels were also increased in the primary cultured WT neurons expressing SOD1G93A-RFP, compared with WT neurons expressing SOD1WT-RFP (Figure S8b). Thus, the cytoplasmic accumulation of SOD1 protein by mutated SOD1 contributes to aggravate ER stress more seriously.

Under high ER stress condition with 5 μM thapsigargin, severe DNA damage was detected in most WT neurons expressing SOD1G93A-GFP, but somehow the reduced DNA damage was examined in WT neuron expressing SOD1WT-GFP (Figure 3c). This suggests that the nucleic import of SOD1 protein could eventually reduce the DNA damage by reducing ER stress. Thus, restricted cytoplasmic localization of SOD1WT by the SOD1 mutation and being more susceptible to induced ER stress leads to DNA damage via synergistic defects. Interestingly, thapsigargin treatment in the neurons prohibited nucleic translocation of WT and mutated SOD1 proteins (Figure S9). It shows that enhancing ER stress interferes more severely with the nucleic transport of SOD1 proteins and further accentuates and accelerates ER stress. Thus, weakly induced ER stress in SOD1-mutated neurons could induce DNA damage within a short time via accumulated ER stress; the synergistic defect and susceptibility to ER stress result in neuronal death.

### 3.4. SOD1G93A Inhibits the Nucleic Localization of fALS-Related RNA-Binding Protein, FUS, and FUS-Related DNA Repair Enzymes

**Figure 4:**
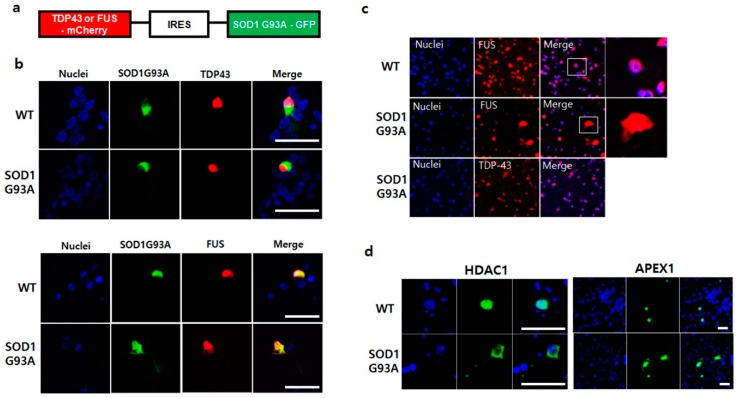
SOD1G93A interfered the nucleic localization of FUS. (**a**) The manipulated plasmid construct for co-expression of SOD1G93A with TDP-43 or FUS protein. (**b**) Mislocalization of FUS protein in the presence of SOD1G93A. The nucleic localization of TDP-43-mCherry was not influenced by co-expressing SOD1G93A-GFP in WT and SOD1G93A genotype cells (upper panel). Whereas nucleic localization of FUS-mCherry was interfered with by co-expressing SOD1G93A-GFP in WT and SOD1G93A genotype cells (lower panel). (scale bar is 50 μm). (**c**) Mislocalized FUS protein in neurons expressing SOD1G93A. FUS protein was normally localized in the nuclei of spinal cord neurons of WT mice (upper). However, the mislocalized FUS proteins were detected in the cytoplasm of neurons from the dissected spinal cords of SOD1G93A genotype mice, unlike nucleic localization of TDP-43 protein in the dissected spinal cord neurons of SOD1G93A genotype mice (lower). (**d**) Mislocalization of HDAC1 and APEX1 in SOD1G93A-expressing neurons. GFP-HDAC1 (left panel) and GFP-APEX1 (right panel) were mislocalized in SOD1G93A genotype neurons (lower) unlike nucleic localization in normal neurons (upper). (scale bar is 50 μm).

The function of cytoplasmic segregated p53 protein in SOD1G93A genotype neurons restrained the DNA repair process (Figure 2b) [39]. Generally, ER stress inhibits the DNA repair process and thus, increases DNA strand breaks [40,41]. Two ALS inducible RNA-binding proteins, TDP43 and FUS, participate in the DNA repair process [42]. TDP-43 is a critical factor required for DNA to repair the nonhomologous end joining (NHEJ)-mediated DNA double-strand break (DSB) in nuclei [43]. Whereas FUS is required for activation of the XRCC1/LigIII ligase to repair DNA nicks in motor neurons [44]. A recent study showed the association between SOD1 and mutated TDP-43 or FUS, wherein the mutated TDP-43 or FUS generates the misfolded SOD1WT protein during fALS progression [20,21]. To understand how SOD1 mutation-linked ER stress prohibits DNA repair, the normal RNA-binding proteins, TDP43-GFP and FUS-GFP, were expressed in the primary cultured WT and SOD1G93A genotype neurons, respectively. Unlike the successful nuclear localization of TDP-43-GFP in WT, the translocation of FUS-GFP into the nucleus was slightly inhibited in SOD1G93A genotype neurons (Figure S10). To further confirm the mislocalization of FUS in the presence of SOD1G93A, we manipulated the plasmid co-expressing with an equal amount of SOD1G93A and TDP-43 or FUS. SOD1G93A-GFP and TDP-43-mCherry or FUS-mCherry were linked together via the IRES sequence to ensure equal expression of both proteins (Figure 4a). When the manipulated plasmid was transfected into both primary cultured WT and SOD1G93A genotype neurons, different localization patterns were observed. TDP-43-mCherry coexpressed with SOD1G93A-GFP was successfully transported into the nucleus in both WT and SOD1G93A genotype neurons without being restricted to the cytoplasm. However, nucleic translocation of FUS-mCherry co-expressed with SOD1G93A-GFP was partially inhibited in both neurons, and the inhibition of nucleic translocation for FUS-mCherry was more significant in SOD1G93A genotype than WT neurons (Figure 4b). Moreover, we further examined the FUS protein from the dissected spinal cords of WT and SOD1G93A genotype mice. FUS proteins were detected in the whole area, including the nucleus in SOD1G93A genotype neurons, different from that in WT neurons, unlike the nucleic localization of all TDP-43 proteins (Figure 4c). Thus, the presence of SOD1G93A only interfered with the nuclear translocation of FUS, suggesting that mutated SOD1 only affects the folding of FUS protein, but not TDP-43 [21]. The cytoplasmic restriction of FUS interfered with DNA repair in SOD1G93A genotype neurons, resulting in the accumulation of damaged DNAs.

The DNA repair process by FUS usually requests various components. It has been reported that the histone deacetylase 1 (HDAC1), that FUS directly interacts with, is required for repair of DEBs in the primary mouse cortical neurons [45]. Poly (ADP-ribose) polymerase (PARP) is also required to collaborate with FUS to prevent ALS progression, and thus, the mislocalization of mutated FUS impairs PARP-dependent DDR signaling [46]. We tested the dysfunctional effect of SOD1G93A on DNA repair enzymes, including FUS-related DNA repair enzymes, such as 8-oxoguanine DNA glycosylase-1 (OGG1), apurinic/apyrimidinic endodeoxyribonuclease 1 (APEX1), HDAC1, PARP1, and X-ray repair cross complementing 1 (XRCC1). The mislocalization of GFP-tagged HDAC1 and APEX1 was clearly observed in many SOD1G93A genotype neurons, but not in WT neurons (Figure 4d). In contrast, the localization of XRCC1, OGG1, and PARP1 in SOD1G93A genotype neurons was similar to that in WT neurons, suggesting that the SOD1 mutation did not affect their localization (Figure S11). Our study showed that cytoplasmic segregation of HDAC1 and APEX1 interfered the normal DNA repair process in SOD1G93A-expressing neurons; thus, the mislocalization of certain DNA repair process-related proteins and enzymes could directly enhance DNA damage in SOD1-mutation linked ALS.

### 3.5. Overexpressed PDI Enhances Nuclear Translocation of SOD1G93A

**Figure 5:**
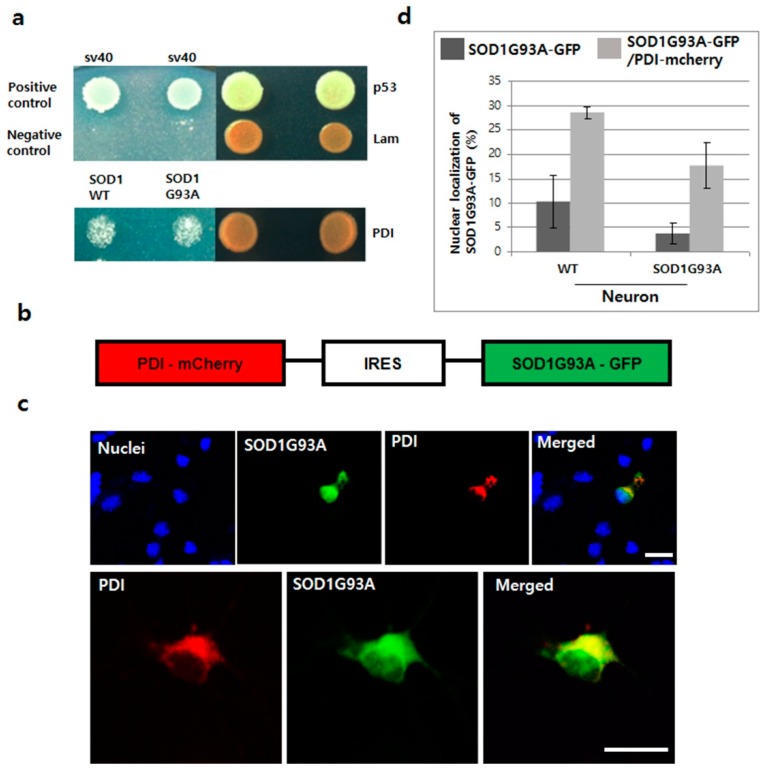
Translocation of SOD1G93A into nuclei by overexpressing PDI in primary cultured neurons. (**a**) Interaction between SOD1G93A and PDI. SOD1WT and SOD1G93A interaction with PDI in the yeast two-hybrid system. (**b**) Plasmid constructed for co-expressing SOD1G93A-GFP and PDI-mCherry. (**c**) Nucleic localization of SOD1G93A with PDI. The co-expressed PDI-mCherry (red) enhanced the nucleic localization of SOD1G93A-GFP (green) in primary cultured neurons (upper). Both SOD1G93A-GFP and PDI-mCherry proteins were colocalized in the ER, whereas, only SOD1G93A-GFP was detected in the nuclei (lower). (scale bar is 20 μm). (**d**) Statistical analysis of nucleic localization of SOD1G93A-GFP in primary cultured WT and SOD1G93A neurons (results in triplicates); Dark gray color: Only SOD1G93A-GFP expression; weak gray color: Co-expression of SOD1G93A-GFP and PDI-mCherry. (*n* = 116 for WT neurons and 157 for SOD1G93A background neurons, error bars: Standard deviation).

Protein disulphide isomerase (PDI) functions as a protective molecule in ALS, by refolding the misfolded proteins within the ER. For example, overexpressed PDI increases antibody secretion by regenerating the disulfide bond [47,48]. Upregulated PDI reduces the aggregation of mutated SOD1 and cell apoptosis, as seen in the spinal cords of SOD1G93A TG mice and rats and in human ALS postmortem tissue [49]. In our yeast two-hybrid test, PDI directly interacted with both SOD1WT and G93A, suggesting that the misfolded structure of SOD1 could be corrected by the chaperone function of PDI (Figure 5a). Accordingly, we anticipated that the chaperone function of PDI could also help to translocate the mutated SOD1 protein after refolding, and hence, manipulated the plasmid co-expressing with PDI and SOD1G93A, connected via IRES (Figure 5b). When PDI-mCherry was co-expressed with SOD1G93A-GFP in both WT and SOD1G93A genotype neurons, nucleic importation of SOD1G93A-GFP was clearly observed along with cytoplasmic overexpression of PDI protein (Figure 5c). In statistical analysis, the nucleic localization of SOD1G93A-GFP was significantly increased in neurons co-expressing with PDI-mCherry, about three times more than that in WT neurons, which only expressed SOD1G93A-GFP (Figure 5d). Our experimental results confirm that PDI overexpression enhances the translocation of mutated SOD1 and reduces ER stress by removing the aggregated SOD1 protein from the cytoplasm [49].

Next, we examined the effect of overexpressed PDI on reducing neuronal death in NSC34 cells. First, we confirmed that the nucleic localization of cytoplasmic segregated SOD1G93A-GFP was enhanced in NSC34 cells by co-expressing with PDI-mCherry (Figure S12a). SOD1G93A genotype neurons are more susceptible to ER stress inducers and usually exhibit a high percentage of cell death (Figure S7). To test the effect of PDI overexpression on rescuing neuronal death, two different NSC34 cell types were sorted out using FACS: One type is cells that only expressed SOD1G93A-GFP, and the other type that co-expressed SOD1G93A-GFP and PDI-mCherry. In cellular apoptosis experiments with Annexin V as a cell apoptosis marker, after treating with 1 μM thapsigargin for 12 h, around 18% cells expressing SOD1G93A-GFP progressed to apoptosis, whereas, only 8% cells co-expressing SOD1G93A-GFP and PDI-mCherry, the reduced cell apoptosis was examined. Thus, co-expression of PDI-mCherry in SOD1G93A-GFP-expressed neurons significantly reduced neuronal death (Figure S12b), which indicates that PDI overexpression could rescue the transport of cytoplasm-localized SOD1G93A into the nucleus, resulting in a reduction of ER stress and DNA damage, thereby reducing ER-stress induced neuronal death.

## 4. Discussion

The connection of SOD1 mutation with DNA damage has been debatable, thus far. Some researchers believe that SOD1 mutation is not associated with DNA damage, as it does not increase DNA breaks in the spinal cord of SOD1G93A genotype TG mice [22]. Unlike in the primary cultured neurons, ATM and its substrate p-H2Ax, both acting as DNA damage markers, were upregulated in the neurons from the spinal cord of 70-day-old SOD1G93A TG mice, along with increased levels of p53 protein, which participates in the DNA repair process (Figure 2). Clearly, our results indicate that SOD1 mutation directly enhances DNA damage during aging. Interestingly, neuronal apoptosis assay using primary cultured neurons showed no significant differences in defects between WT and SOD1G93A genotype neurons, when treated with oxidative stress inducer, H_2_O_2_, different from treatment with ER stress inducer, thapsigargin (Figures S6 and S7). This shows that DNA damage-linked neuronal death in SOD1 mutated neurons was more susceptible to ER stress than oxidative stress, provided the DNA damage progresses the same way in both SOD1G93A genotype and WT neurons (Figure S5). These results suggest that DNA damage induced by SOD1 mutation is closely related to gain-of-function ER toxicity, instead of loss of antioxidant function in SOD-mutations linked to ALS [9,50].

**Figure 6:**
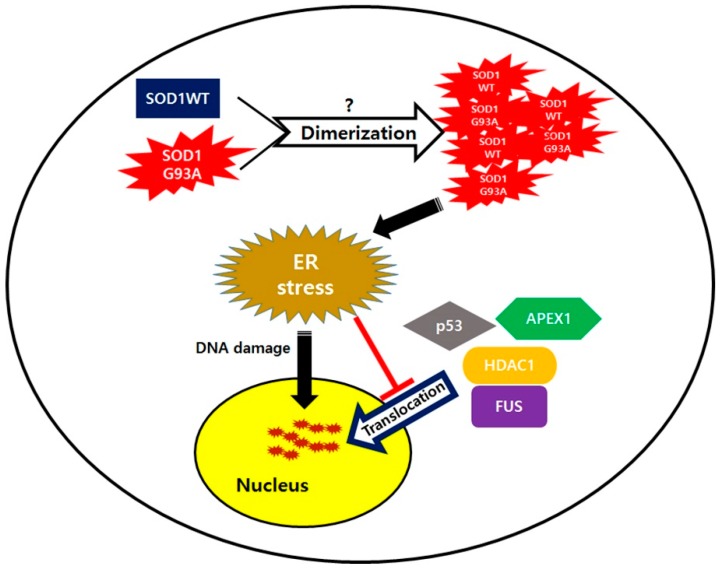
SOD1 mutation mediated amyotrophic lateral sclerosis (ALS) pathogenic progression model.

A previous study showed that ALS-linked mutated proteins interact with normal proteins, which is also subsequently involved in ALS inducement, and their possible interaction leads to misfolding of normal ALS-linked proteins. For example, mutated FUS in fALS and the pathogenic form of TDP-43 in sALS, both can interact with SOD1WT and change its normal structure into the pathogenic form by misfolding SOD1WT [21]. Then, the misfolded SOD1WT protein, acting as an initiator, propagates itself and aggravates ALS progression [20]. Our results were consistent with this theory, that SOD1WT function is influenced by the mutated SOD1. SOD1WT failed to translocate into the nuclei in the presence of cytoplasmic sequestered SOD1G93A (Figure 1b). Generally, the dimeric formation of normal SOD1 is essential to maintain antioxidant enzyme function [51]. However, in heterodimer formation of SOD1, mutated SOD1 could be generated from mutated single copy gene in neurons and in the heterodimer form, the structure of SOD1WT is gradually changed to the misfolded form when interacting with the mutated SOD1 protein, similar to that seen in prion propagation (Figure 6). In SOD1 mutant-mediated ALS progression, the heterodimer form of the normal and mutated SOD1, which generates oligomers in neurons via the exposed cross-linked disulfide bonds, could be a more serious cause in neuronal damages [52]. The failed nuclear translocation of SOD1WT is consistent with other studies, reporting that the pathogenic process of ALS has very similar characteristics as prion disease progression [53,54]. A normal prion protein PrP^C^ could transit into the misfolded pathogenic form PrP^Sc^ without gene mutation. The pathogenic PrP^Sc^ is self-propagating and transmissible when interacting with normal PrP^C^ [55,56]. Similarly, mutated SOD1 could also progress the conformational change of normal SOD1 protein to the pathogenic form during dimer formation (Figure 6). In that case, the resulted dimer eventually aggregates to the cytoplasmically segregated SOD1 proteins, similar to plaque formation in prion disease neurons [57]. The C-terminal segment of SOD1, believed to play a role in nucleation and fibril growth, participates in aggregation and generates large SOD1 oligomers that fail to transport into the nucleic pore complex (NPC), thereby resulting in segregated SOD1 proteins restricted to the cytoplasm [58].

In our study, ALS linked RNA binding protein, normal FUS, was restricted to the cytoplasm in the presence of SOD1G93A (Figure 4b). In contrast to a previous study wherein mutated FUS and TDP-43 were shown to change the structure of SOD1WT [20], the observed cytosolic segregated FUS indicated that the SOD1G93A could also misfold the normal FUS protein, but not TDP-43. Thus, ALS linked mutation of SOD1 and FUS exhibited mutual interactions such that the mutated protein always misfolded the normal protein simultaneously (Figure 4b). Similar to previous reports, SOD1G93A inhibiting nucleic translocation of FUS suggested that the heterodimeric formation between different protein types may lead to pathogenic conformational changes, crucial for the initiation and progression of ALS disease.

In ALS, DNA damage is observed in the motor neurons, and as a result, various nucleic DNA repair enzymes, such as PARP1, OGG1, and APEX1 are generally upregulated in the ALS mouse model and patient samples [59,60,61]. Despite the enhanced expressions of DNA repair enzymes, DNA damage still accumulated in neurons of the spinal cord of SOD1G93A TG mice (Figure 2a and Figure S4), suggesting that the DNA repair process was not normally accomplished. Unlike other tested DNA repair enzymes, the cytosolic segregation of APEX1 was observed in many SOD1G93A-expressing neurons showing deficient DNA repair process. Thus, we believe that cytosolic mislocalization of APEX1 is the main reason for accumulated DNA damage in neurons expressing SOD1G93A despite upregulation of DNA repair enzymes (Figure 4d). Moreover, several mutations of the APEX1 gene were also observed in ALS patients, suggesting that APEX1 dysfunction is directly associated with ALS occurrence [62,63]. Clearly, restricted localization of APEX1 in the cytoplasm has the same effect on ALS occurrence as loss of function by gene mutation. In addition, ALS-linked FUS was mislocalized in the cytoplasm of neurons expressing SOD1G93A (Figure 4b,c). Previous reports show that PARP1 and HDAC1 are required for recruiting FUS protein into the damaged DNA, and DNA repair process can only be accomplished by establishing a direct interaction between FUS and HDAC1 [45,64]. In our study, the SOD1G93A-expressing neurons showed that HDAC1 localization was also segregated instead of nucleic translocation, but nucleic localization of PARP1 was clearly observed (Figure 4d and Figure S10). Thus, we believe that cytoplasmic segregation of FUS in SOD1G93A genotype neurons is closely related to the cytosolic mislocalization of HDAC1 (Figure 6).

In an environment of ER stress, the DNA repair process generally slows down as the level of DNA repair enzymes decreases, and thus, DNA damage is enhanced [40]. Compared to WT neurons, SOD1G93A-expressing neurons are more susceptible to the ER stress inducer, resulting in dramatic cytosolic Ca^2+^ increase and PDI upregulation, owing to the cytosolic segregated SOD1G93A proteins (Figure S7a,b). Our result shows that increasing ER stress restricts the nucleic transportation of SOD1 proteins, leading to accumulating SOD1 protein in the cytoplasm (Figure S8). Therefore, nucleic localization for DNA repair-related proteins, such as APEX1, HDAC1, and FUS, is more severely blocked by the enhanced ER stress (Figure 6). Therefore, enhanced ER stress increases inhibition of nucleic translocation of DNA repair enzymes. Generally, the subcellular localization is very important for proteins to maintain their original functions, and thus, the mislocalization of DNA repair proteins retards their efficiency in the DNA repair process. Eventually, DNA damage in SOD1G93A genotype neurons is accelerated via the reduced number of nuclear DNA repair enzymes.

In the motor neurons of SOD1G93A genotype spinal cord, nucleic localization of upregulated p53 was also inhibited, due to increased ER stress (Figure 2b). In response to DNA damage, p53 as the master protein plays various roles in a variety of DNA-damage-response mechanisms, and directly influences the activity of various DNA-repair systems. Nucleic translocation of p53 is required to bind to DNA, upregulate the transcription of DNA repair protein genes, and significantly facilitate DNA repair [39]. When localization of activated p53 protein responding to the DNA damage is restricted to the cytoplasm or the p53 protein is degraded by E3 ubiquitin-ligase under ER stress, its function in the DNA repair process is seriously affected [36,65]. In our experiment with the spinal cord neurons of SOD1G93A TG mice, the cytoplasmic localization of p53 was significantly increased with enhanced ER stress, but its upregulation in western blotting suggests that p53 degradation probably is a cell-type-specific dependent process (Figure 2c). Thus, DNA damage increases in the spinal cord neurons of the spinal cord of SOD1G93A TG mouse mice is due to p53 localization and DNA repair enzymes both restricted to the cytoplasm is restricted localization of p53, as well as DNA repair enzymes (Figure 2a and Figure S4).

Nucleic transport of SOD1 is directly associated with DNA damage under ER stress (Figure 3c). PDI, which activates the nucleic translocation of SOD1G93A, is very important to decrease ER stress and thus, reduce DNA damage in neurons expressing SOD1G93A (Figure 5c,d). Unfortunately, primary cultured SOD1G93A genotype neurons are not the most suitable samples to study the protective function of PDI on DNA damage, because it is impossible to artificially induce DNA damage and overexpress PDI evenly in all neurons. In the glioblastoma multiform tumor model, PDI is directly associated with DNA repair process by maintaining a specific level of DNA repair enzymes [66,67]. Thus, increasing PDI that activates the SOD1G93A protein transport into the nucleus could enhance the nucleic translocation of DNA repair enzymes by reducing ER stress. Moreover, the nucleic translocation of SOD1G93A by overexpressed PDI could also reduce cellular apoptosis under high ER stress (Figure S11b). As previously reported, the interaction of mutant SOD1 with Derlin-1 leads to increased apoptosis by activating ER stress-induced apoptosis signal-regulating kinase 1 (ASK1) [68]. Thus, expression of PDI, which corrects the misfolded structure of SOD1G93A protein via direct interaction, could break down the interaction of SOD1G93A with Derlin-1, inactivate ASK1, and reduce ER stress (Figure 5a). In addition, PDI protein is also required to stabilize p53, which is involved in the protection system to prevent cell apoptosis [69]. Thus, overexpressing PDI could stabilize p53 and enhance its activity to reduce cellular apoptosis and DNA damage under high ER stress. However, further studies are still required to provide more detailed information.

## 5. Conclusions

In studies, restricted localization of SOD1G93A in the cytoplasm resulted in DNA damage in the spinal cord motor neurons during aging. With cell biological and biochemical approaches, we discovered that the cytosolic presence of SOD1G93A interfered with the nucleic transport of SOD1WT, as well as DNA repair processing-related proteins, such as p53, FUS, HDAC1, and APEX1. These cytosolic restrained and segregated proteins further enhanced ER stress and eventually induced DNA damage. Thus, DNA damage in ALS-linked SOD1 mutation might be closely related to the dysfunction of DNA repair process during aging. We have also shown that ectopic expression of PDI, one of the chaperone proteins, translocated the SOD1G93A into the nucleus, rescued its cytosolic mislocalization, and protected neuronal death under high ER stress, suggesting the possible connection between SOD1G93A mislocalization and neuronal death in SOD1 mutation-linked ALS.

## Supplementary Materials

The following are available online at https://www.mdpi.com/2073-4409/8/12/1502/s1, Figure S1: Result of FACS analysis for transfection of plasmids into primary cultured mouse neuron (left) and NSC34 cells (right), Figure S2: Different localization between SOD1WT and SOD1G93A, Figure S3: Measurement of RNA level of GFP and RFP region in the constructed plasmid using RT-qPCR, Figure S4: Detection of ATM in WT and SOD1G93A in spinal cords dissected from the WT and SOD1G93A transgenic mice aged 70 days, Figure S5: Degradation of genomic DNA with treatment of 0.6 mM H_2_O_2_ and 1 μM thapsigargin, Figure S6: H_2_O_2_ exhibited no effect on inducing cell apoptosis, Figure S7: Enhanced cell death in SOD1G93A genotype neurons under ER stress, Figure S8: Increase the ER stress markers in SOD1G93A genotype neurons with treatment of 1 μM thapsigargin, Figure S9: Inhibition of nucleic translocation of SOD1 protein under ER stress, Figure S10: Localization of TDP-43 and FUS proteins in SOD1WT and SOD1G93A genotype neurons, Figure S11: Nucleic localization of XRCC and OGG1, Figure S12: Transport of SOD1G93A into nuclei and rescue of cellular apoptosis by PDI overexpression in NSC34 cells.

## References

[1] Yoshida S., Mulder D.W., Kurland L.T., Chu C.P., Okazaki H. (1986). Follow-up study on amyotrophic lateral sclerosis in Rochester, Minn., 1925 through 1984. Neuroepidemiology.

[2] Haverkamp L.J., Appel V., Appel S.H. (1995). Natural history of amyotrophic lateral sclerosis in a database population. Validation of a scoring system and a model for survival prediction. Brain.

[3] Nelson L.M. (1995). Epidemiology of ALS. Clin. Neurosci..

[4] Pasinelli P., Brown R.H. (2006). Molecular biology of amyotrophic lateral sclerosis: Insights from genetics. Nat. Rev. Neurosci..

[5] Gros-Louis F., Gaspar C. (2006). Rouleau GA: Genetics of familial and sporadic amyotrophic lateral sclerosis. Biochim. Biophys. Acta.

[6] Taylor J.P., Brown R.H., Cleveland D.W. (2016). Decoding ALS: From genes to mechanism. Nature.

[7] Deng H.X., Hentati A., Tainer J.A., Iqbal Z., Cayabyab A., Hung W.Y., Getzoff E.D., Hu P., Herzfeldt B., Roos R.P. (1993). Amyotrophic lateral sclerosis and structural defects in Cu,Zn superoxide dismutase. Science.

[8] Rosen D.R., Siddique T., Patterson D., Figlewicz D.A., Sapp P., Hentati A., Donaldson D., Goto J., O’Regan J.P., Deng H.X. (1993). Mutations in Cu/Zn superoxide dismutase gene are associated with familial amyotrophic lateral sclerosis. Nature.

[9] Sau D., De Biasi S., Vitellaro-Zuccarello L., Riso P., Guarnieri S., Porrini M., Simeoni S., Crippa V., Onesto E., Palazzolo I. (2007). Mutation of SOD1 in ALS: A gain of a loss of function. Hum. Mol. Genet..

[10] Mackenzie I.R., Rademakers R., Neumann M. (2010). TDP-43 and FUS in amyotrophic lateral sclerosis and frontotemporal dementia. Lancet Neurol..

[11] Guerrero E.N., Wang H., Mitra J., Hegde P.M., Stowell S.E., Liachko N.F., Kraemer B.C., Garruto R.M., Rao K.S., Hegde M.L. (2016). TDP-43/FUS in motor neuron disease: Complexity and challenges. Prog. Neurobiol..

[12] Kim S.H., Shanware N.P., Bowler M.J., Tibbetts R.S. (2010). Amyotrophic lateral sclerosis-associated proteins TDP-43 and FUS/TLS function in a common biochemical complex to co-regulate HDAC6 mRNA. J. Biol. Chem..

[13] Yamazaki T., Chen S., Yu Y., Yan B., Haertlein T.C., Carrasco M.A., Tapia J.C., Zhai B., Das R., Lalancette-Hebert M. (2012). FUS-SMN protein interactions link the motor neuron diseases ALS and SMA. Cell Rep..

[14] Ling S.C., Albuquerque C.P., Han J.S., Lagier-Tourenne C., Tokunaga S., Zhou H., Cleveland D.W. (2010). ALS-associated mutations in TDP-43 increase its stability and promote TDP-43 complexes with FUS/TLS. Proc. Natl. Acad. Sci. USA.

[15] Lanson N.A., Maltare A., King H., Smith R., Kim J.H., Taylor J.P., Lloyd T.E., Pandey U.B. (2011). A Drosophila model of FUS-related neurodegeneration reveals genetic interaction between FUS and TDP-43. Hum. Mol. Genet..

[16] Kabashi E., Bercier V., Lissouba A., Liao M., Brustein E., Rouleau G.A., Drapeau P. (2011). FUS and TARDBP but not SOD1 interact in genetic models of amyotrophic lateral sclerosis. PLoS Genet..

[17] Sturtz L.A., Diekert K., Jensen L.T., Lill R., Culotta V.C. (2001). A fraction of yeast Cu, Zn-superoxide dismutase and its metallochaperone, CCS, localize to the intermembrane space of mitochondria. A physiological role for SOD1 in guarding against mitochondrial oxidative damage. J. Biol. Chem..

[18] Belzil V.V., Daoud H., Dion P.A., Rouleau G.A. (2011). No effect on SOD1 splicing by TARDP or FUS mutations. Arch. Neurol..

[19] Somalinga B.R., Day C.E., Wei S., Roth M.G., Thomas P.J. (2012). TDP-43 identified from a genome wide RNAi screen for SOD1 regulators. PLoS ONE.

[20] Pokrishevsky E., Grad L.I., Cashman N.R. (2016). TDP-43 or FUS-induced misfolded human wild-type SOD1 can propagate intercellularly in a prion-like fashion. Sci. Rep..

[21] Pokrishevsky E., Grad L.I., Yousefi M., Wang J., Mackenzie I.R., Cashman N.R. (2012). Aberrant localization of FUS and TDP43 is associated with misfolding of SOD1 in amyotrophic lateral sclerosis. PLoS ONE.

[22] Penndorf D., Tadic V., Witte O.W., Grosskreutz J., Kretz A. (2017). DNA strand breaks and TDP-43 mislocation are absent in the murine hSOD1G93A model of amyotrophic lateral sclerosis in vivo and in vitro. PLoS ONE.

[23] Grad L.I., Guest W.C., Yanai A., Pokrishevsky E., O’Neill M.A., Gibbs E., Semenchenko V., Yousefi M., Wishart D.S., Plotkin S.S. (2011). Intermolecular transmission of superoxide dismutase 1 misfolding in living cells. Proc. Natl. Acad. Sci. USA.

[24] Cashman N.R., Durham H.D., Blusztajn J.K., Oda K., Tabira T., Shaw I.T., Dahrouge S., Antel J.P. (1992). Neuroblastoma x spinal cord (NSC) hybrid cell lines resemble developing motor neurons. Dev. Dyn..

[25] Al-Chalabi A., Leigh P.N. (2000). Recent advances in amyotrophic lateral sclerosis. Curr. Opin. Neurol..

[26] Shukla V., Mishra S.K., Pant H.C. (2011). Oxidative stress in neurodegeneration. Adv. Pharm. Sci..

[27] Murata T., Ohtsuka C., Terayama Y. (2008). Increased mitochondrial oxidative damage and oxidative DNA damage contributes to the neurodegenerative process in sporadic amyotrophic lateral sclerosis. Free Radic. Res..

[28] Aguirre N., Beal M.F., Matson W.R., Bogdanov M.B. (2005). Increased oxidative damage to DNA in an animal model of amyotrophic lateral sclerosis. Free Radic. Res..

[29] Burma S., Chen B.P., Murphy M., Kurimasa A., Chen D.J. (2001). ATM phosphorylates histone H2AX in response to DNA double-strand breaks. J. Biol. Chem..

[30] Shieh S.Y., Ikeda M., Taya Y., Prives C. (1997). DNA damage-induced phosphorylation of p53 alleviates inhibition by MDM2. Cell.

[31] Banin S., Moyal L., Shieh S., Taya Y., Anderson C.W., Chessa L., Smorodinsky N.I., Prives C., Reiss Y., Shiloh Y. (1998). Enhanced phosphorylation of p53 by ATM in response to DNA damage. Science.

[32] Canman C.E., Lim D.S., Cimprich K.A., Taya Y., Tamai K., Sakaguchi K., Appella E., Kastan M.B., Siliciano J.D. (1998). Activation of the ATM kinase by ionizing radiation and phosphorylation of p53. Science.

[33] Barbosa L.F., Cerqueira F.M., Macedo A.F., Garcia C.C., Angeli J.P., Schumacher R.I., Sogayar M.C., Augusto O., Carri M.T., Di Mascio P. (2010). Increased SOD1 association with chromatin, DNA damage, p53 activation, and apoptosis in a cellular model of SOD1-linked ALS. Biochim. Biophys. Acta.

[34] Moll U.M., Laquaglia M., Benard J., Riou G. (1995). Wild-Type P53 Protein Undergoes Cytoplasmic Sequestration in Undifferentiated Neuroblastomas but Not in Differentiated Tumors. Proc. Natl. Acad. Sci. USA.

[35] Kikuchi H., Almer G., Yamashita S., Guegan C., Nagai M., Xu Z.S., Sosunov A.A., McKhann G.M., Przedborski S. (2006). Spinal cord endoplasmic reticulum stress associated with a microsomal accumulation of mutant superoxide dismutase-1 in an ALS model. Proc. Natl. Acad. Sci. USA.

[36] Qu L.K., Huang S., Baltzis D., Rivas-Estilla A.M., Pluquet O., Hatzoglou M., Koumenis C., Taya Y., Yoshimura A., Koromilas A.E. (2004). Endoplasmic reticulum stress induces p53 cytoplasmic localization and prevents p53-dependent apoptosis by a pathway involving glycogen synthase kinase-3 beta. Gene Dev..

[37] Dicks N., Gutierrez K., Michalak M., Bordignon V., Agellon L.B. (2015). Endoplasmic reticulum stress, genome damage, and cancer. Front. Oncol..

[38] Atkin J.D., Farg M.A., Walker A.K., McLean C., Tomas D., Horne M.K. (2008). Endoplasmic reticulum stress and induction of the unfolded protein response in human sporadic amyotrophic lateral sclerosis. Neurobiol. Dis..

[39] Williams A.B., Schumacher B. (2016). p53 in the DNA-Damage-Repair Process. Csh Perspect. Med..

[40] Yamamori T., Meike S., Nagane M., Yasui H., Inanami O. (2013). ER stress suppresses DNA double-strand break repair and sensitizes tumor cells to ionizing radiation by stimulating proteasomal degradation of Rad51. FEBS Lett..

[41] Dicks N., Bohrer R.C., Gutierrez K., Michalak M., Agellon L.B., Bordignon V. (2017). Relief of endoplasmic reticulum stress enhances DNA damage repair and improves development of pre-implantation embryos. PLoS ONE.

[42] Hill S.J., Mordes D.A., Cameron L.A., Neuberg D.S., Landini S., Eggan K., Livingston D.M. (2016). Two familial ALS proteins function in prevention/repair of transcription-associated DNA damage. Proc. Natl. Acad. Sci. USA.

[43] Mitra J., Guerrero E.N., Hegde P.M., Liachko N.F., Wang H.B., Vasquez V., Gao J.L., Pandey A., Taylor J.P., Kraemer B.C. (2019). Motor neuron disease-associated loss of nuclear TDP-43 is linked to DNA double-strand break repair defects. Proc. Natl. Acad. Sci. USA.

[44] Wang H.B., Guo W.T., Mitra J., Hegde P.M., Vandoorne T., Eckelmann B.J., Mitra S., Tomkinson A.E., Van den Bosch L., Hegde M.L. (2018). Mutant FUS causes DNA ligation defects to inhibit oxidative damage repair in Amyotrophic Lateral Sclerosis. Nat. Commun..

[45] Wang W.Y., Pan L., Su S.C., Quinn E.J., Sasaki M., Jimenez J.C., Mackenzie I.R.A., Huang E.J., Tsai L.H. (2013). Interaction of FUS and HDAC1 regulates DNA damage response and repair in neurons. Nat. Neurosci..

[46] Naumann M., Pal A., Goswami A., Lojewski X., Japtok J., Vehlow A., Naujock M., Gunther R., Jin M., Stanslowsky N. (2018). Impaired DNA damage response signaling by FUS-NLS mutations leads to neurodegeneration and FUS aggregate formation. Nat. Commun..

[47] Shusta E.V., Raines R.T., Pluckthun A., Wittrup K.D. (1998). Increasing the secretory capacity of Saccharomyces cerevisiae for production of single-chain antibody fragments. Nat. Biotechnol..

[48] Borth N., Mattanovich D., Kunert R., Katinger H. (2005). Effect of increased expression of protein disulfide isomerase and heavy chain binding protein on antibody secretion in a recombinant CHO cell line. Biotechnol. Prog..

[49] Walker A.K., Farg M.A., Bye C.R., McLean C.A., Horne M.K., Atkin J.D. (2010). Protein disulphide isomerase protects against protein aggregation and is S-nitrosylated in amyotrophic lateral sclerosis. Brain.

[50] Sahin A., Held A., Bredvik K., Major P., Achilli T.M., Kerson A.G., Wharton K., Stilwell G., Reenan R. (2017). Human SOD1 ALS Mutations in a Drosophila Knock-In Model Cause Severe Phenotypes and Reveal Dosage-Sensitive Gain- and Loss-of-Function Components. Genetics.

[51] Valentine J.S., Doucette P.A., Zittin Potter S. (2005). Copper-zinc superoxide dismutase and amyotrophic lateral sclerosis. Annu. Rev. Biochem..

[52] Anzai I., Tokuda E., Mukaiyama A., Akiyama S., Endo F., Yamanaka K., Misawa H., Furukawa Y. (2017). A misfolded dimer of Cu/Zn-superoxide dismutase leading to pathological oligomerization in amyotrophic lateral sclerosis. Protein Sci..

[53] Smethurst P., Sidle K.C.L., Hardy J. (2015). Review: Prion-like mechanisms of transactive response DNA binding protein of 43kDa (TDP-43) in amyotrophic lateral sclerosis (ALS). Neuropathol. Appl. Neurobiol..

[54] Sibilla C., Bertolotti A. (2017). Prion Properties of SOD1 in Amyotrophic Lateral Sclerosis and Potential Therapy. Cold Spring Harb. Perspect. Boil..

[55] Jucker M., Walker L.C. (2013). Self-propagation of pathogenic protein aggregates in neurodegenerative diseases. Nature.

[56] Prusiner S.B. (1982). Novel Proteinaceous Infectious Particles Cause Scrapie. Science.

[57] Munch C., O’Brien J., Bertolotti A. (2011). Prion-like propagation of mutant superoxide dismutase-1 misfolding in neuronal cells. Proc. Natl. Acad. Sci. USA.

[58] Ivanova M.I., Sievers S.A., Guenther E.L., Johnson L.M., Winkler D.D., Galaleldeen A., Sawaya M.R., Hart P.J., Eisenberg D.S. (2014). Aggregation-triggering segments of SOD1 fibril formation support a common pathway for familial and sporadic ALS. Proc. Natl. Acad. Sci. USA.

[59] McGurk L., Mojsilovic-Petrovic J., Van Deerlin V.M., Shorter J., Kalb R.G., Lee V.M., Trojanowski J.Q., Lee E.B., Bonini N.M. (2018). Nuclear poly(ADP-ribose) activity is a therapeutic target in amyotrophic latera sclerosis. Acta Neuropathol. Commun..

[60] Murakami T., Nagai M., Miyazaki K., Morimoto N., Ohta Y., Kurata T., Takehisa Y., Kamiya T., Abe K. (2007). Early decrease of mitochondrial DNA repair enzymes in spinal motor neurons of presymptomatic transgenic mice carrying a mutant SOD1 gene. Brain Res..

[61] Shaikh A.Y., Martin L.J. (2002). DNA base-excision repair enzyme apurinic/apyrimidinic endonuclease/redox factor-1 is increased and competent in the brain and spinal cord of individuals with amyotrophic lateral sclerosis. Neuromol. Med..

[62] Hayward C., Colville S., Swingler R.J., Brock D.J.H. (1999). Molecular genetic analysis of the APEX nuclease gene in amyotrophic lateral sclerosis. Neurology.

[63] Coppede F., Lo Gerfo A., Carlesi C., Piazza S., Mancuso M., Pasquali L., Murri L., Migliore L., Siciliano G. (2010). Lack of association between the APEX1 Asp148Glu polymorphism and sporadic amyotrophic lateral sclerosis. Neurobiol. Aging.

[64] Rulten S.L., Rotheray A., Green R.L., Grundy G.J., Moore D.A.Q., Gomez-Herreros F., Hafezparast M., Caldecott K.W. (2014). PARP-1 dependent recruitment of the amyotrophic lateral sclerosis-associated protein FUS/TLS to sites of oxidative DNA damage. Nucleic Acids Res..

[65] Pluquet O., Qu L.K., Baltzis D., Koromilas A.E. (2005). Endoplasmic reticulum stress accelerates p53 degradation by the cooperative actions of Hdm2 and glycogen synthase kinase 3beta. Mol. Cell. Biol..

[66] Liu Y.J., Ji W.B., Shergalis A., Xu J.Q., Delaney A.M., Calcaterra A., Pal A., Ljungman M., Neamati N., Rehemtulla A. (2019). Activation of the Unfolded Protein Response via Inhibition of Protein Disulfide Isomerase Decreases the Capacity for DNA Repair to Sensitize Glioblastoma to Radiotherapy. Cancer Res..

[67] Xu S.L., Liu Y.J., Yang K., Wang H.X., Shergalis A., Kyani A., Bankhead A., Tamura S., Yang S.H., Wang X. (2019). Inhibition of protein disulfide isomerase in glioblastoma causes marked downregulation of DNA repair and DNA damage response genes. Theranostics.

[68] Nishitoh H., Kadowaki H., Nagai A., Maruyama T., Yokota T., Fukutomi H., Noguchi T., Matsuzawa A., Takeda K., Ichijo H. (2008). ALS-linked mutant SOD1 induces ER stress- and ASK1-dependent motor neuron death by targeting Derlin-1. Gene Dev..

[69] Kranz P., Neumann F., Wolf A., Classen F., Pompsch M., Ocklenburg T., Baumann J., Janke K., Baumann M., Goepelt K. (2017). PDI is an essential redox-sensitive activator of PERK during the unfolded protein response (UPR). Cell Death Dis..

