# Supplementary material for: Cytoplasmic Restriction of Mutated SOD1 Impairs the DNA Repair Process in Spinal Cord Neurons
Source: Cells. 2019 Nov 23;8(12):1502. doi: 10.3390/cells8121502 (PMC6952796; doi:10.3390/cells8121502)
Supplement: Supplementary file 1 [file cells-08-01502-s001.pdf]

Suppl. Figures and figure legends

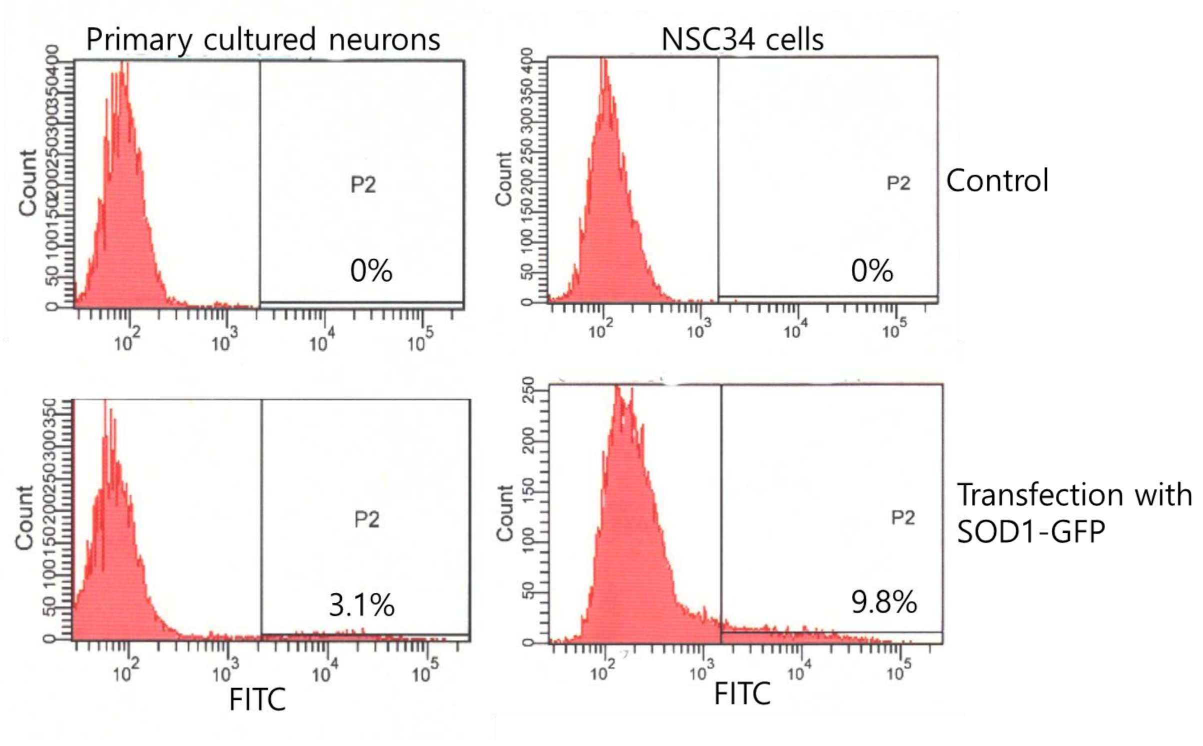

Figure S1. Result of FACS analysis for transfection of plasmids into primary cultured mouse neuron (left) and NSC34 cells (right).

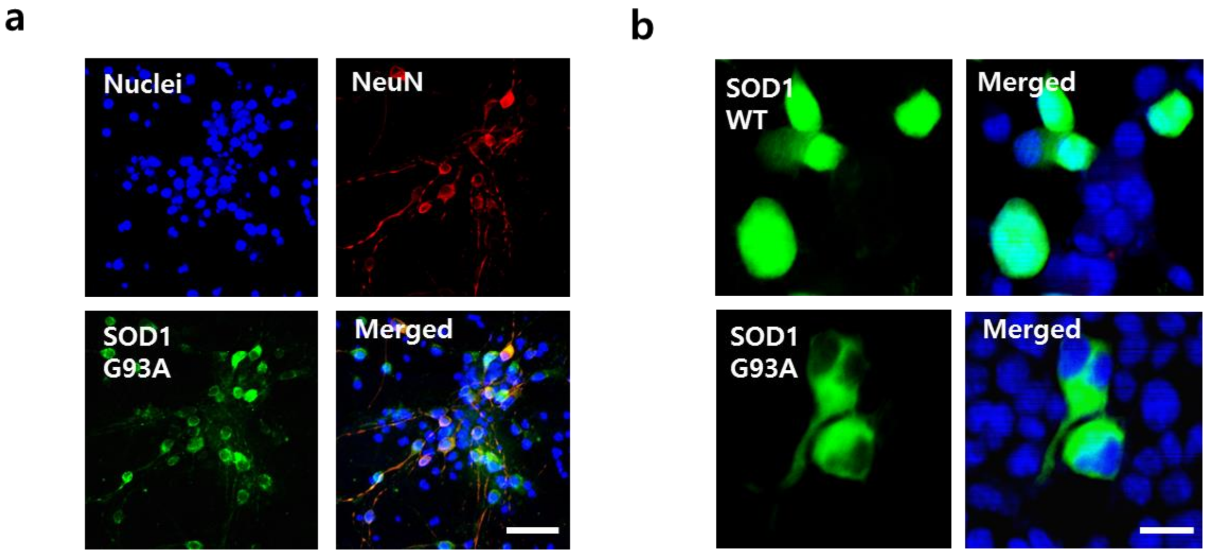

Figure S2. Different localization between SOD1WT and SOD1G93A. (a) hSOD1 G93A (green) staining with SOD1 antibody and MAP2(red) in SOD1 G93A genotype neuron. (scale bar is 10 μm). (b) Localization of SOD1WT-GFP and SOD1G93A-GFP in NSC34 cells. (scale bar is 50 μm).

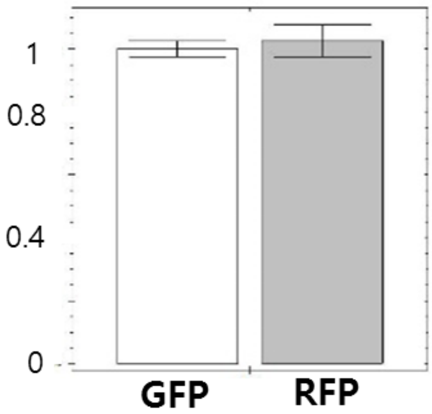

Figure S3. Measurement of RNA level of GFP and RFP region in the constructed plasmid using RT-qPCR. The expressed RNA level of SOD1WT-RFP and SOD1G93A-GFP was same.

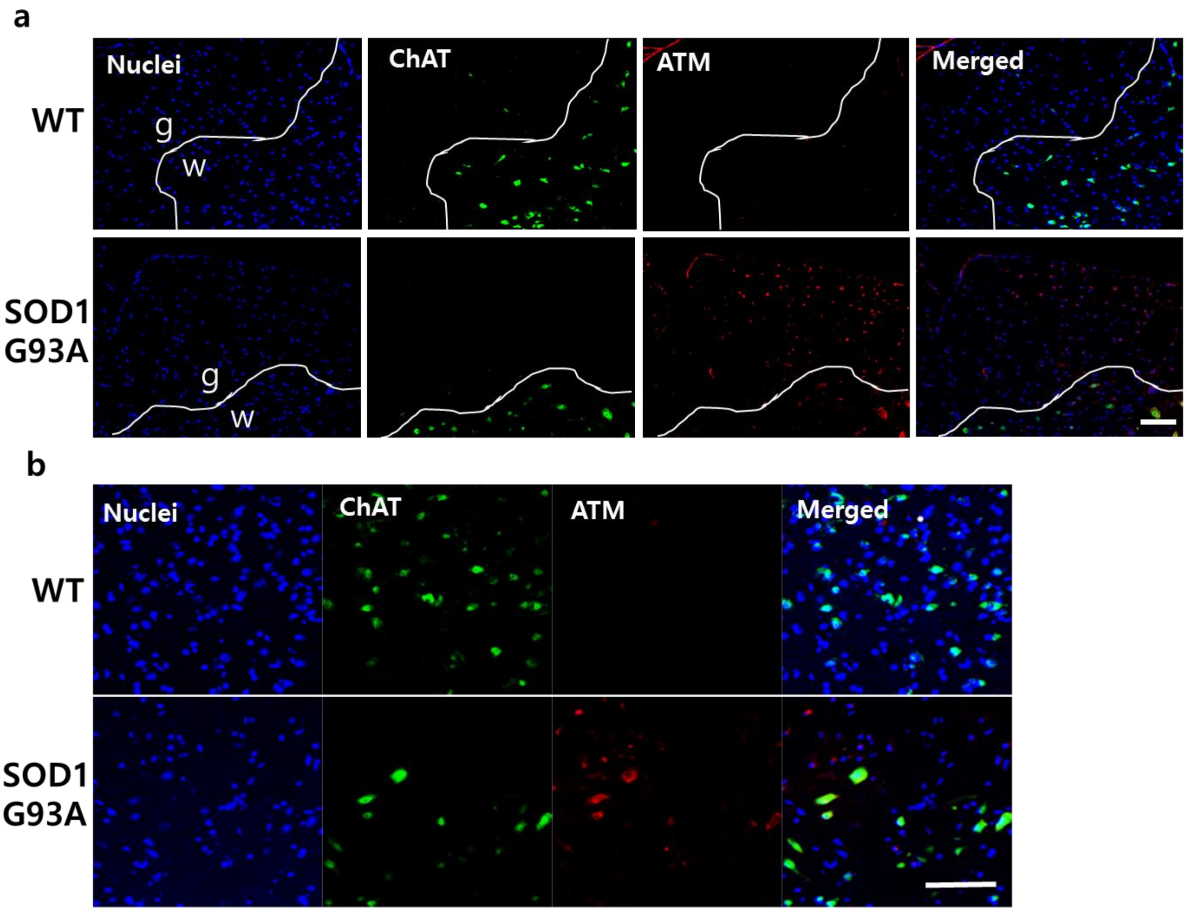

Figure S4. Detection of ATM in WT and SOD1G93A in spinal cords dissected from the WT (upper) and SOD1G93A transgenic mice (down) aged 70 days. (a) The examined ATM in the spinal cord of SOD1G93A transgenic mouse was much more than WT. The divided white and gray regions were indicated as w and g respectively. (scale bar is 100 μm). (b) The detection of ATM marker in the ventral horn region in spinal cord of WT and SOD1G93A transgenic mice. (scale bar is 100 μm).

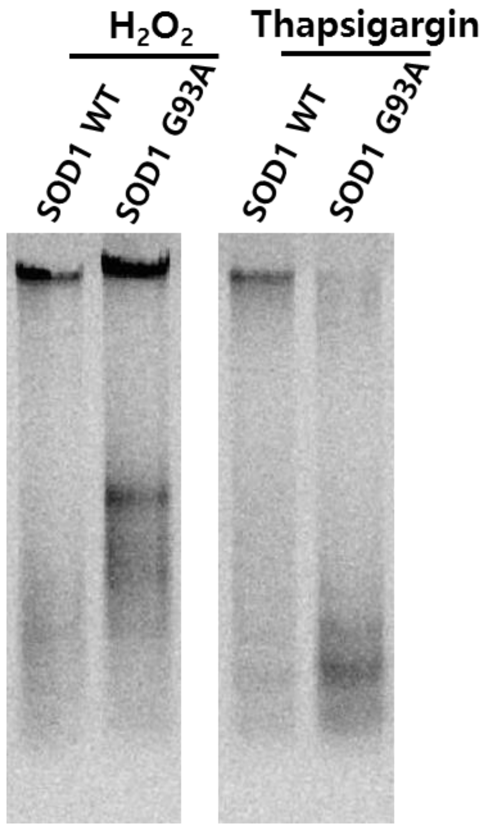

Figure S5. Degradation of genomic DNA with treatment of 0.6 mM H_2O_2 (left) and 1 μM thapsigargin (right). After treatment of 1 μM thapsigargin for three days, genomic DNA in SOD1G93A genotype neurons was completely degraded but not WT (right). However, no difference was noted between the two different genotype neurons under 0.6 mM H_2O_2 treatment for three days (left lane).

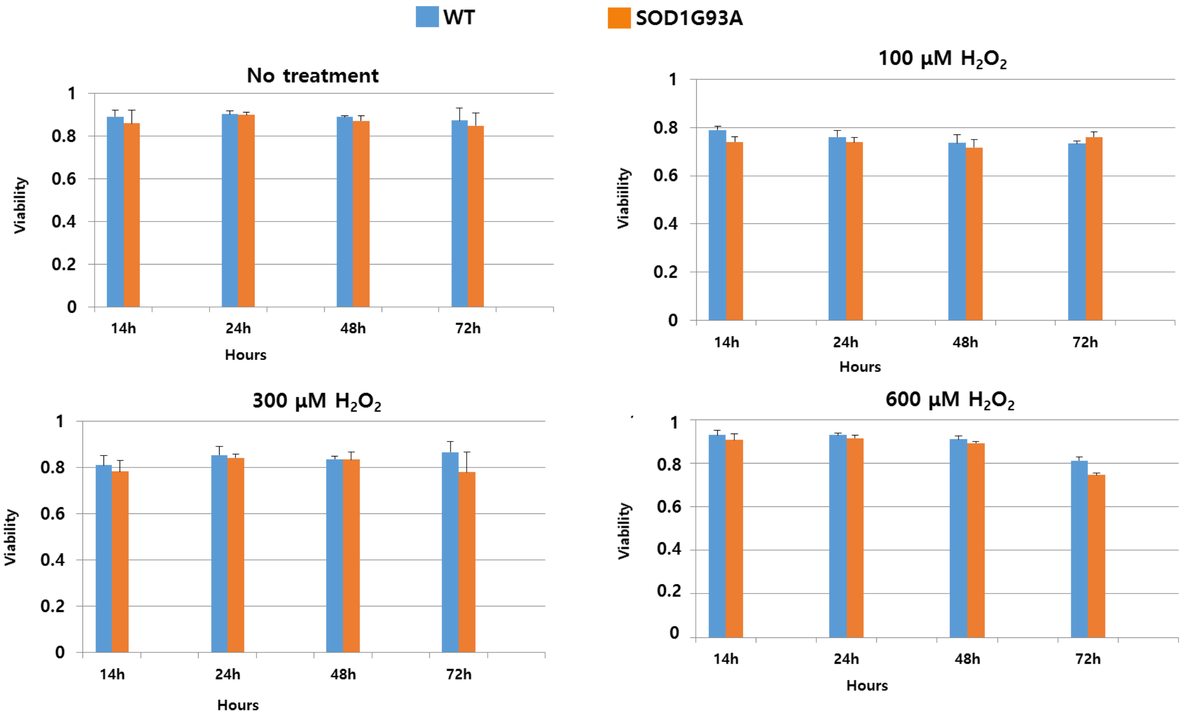

Figure S6. H_2O_2 exhibited no effect on inducing cell apoptosis. Cellular apoptosis in primary cultured neurons was not induced when treated with three different concentration of H_2O_2 for three days.

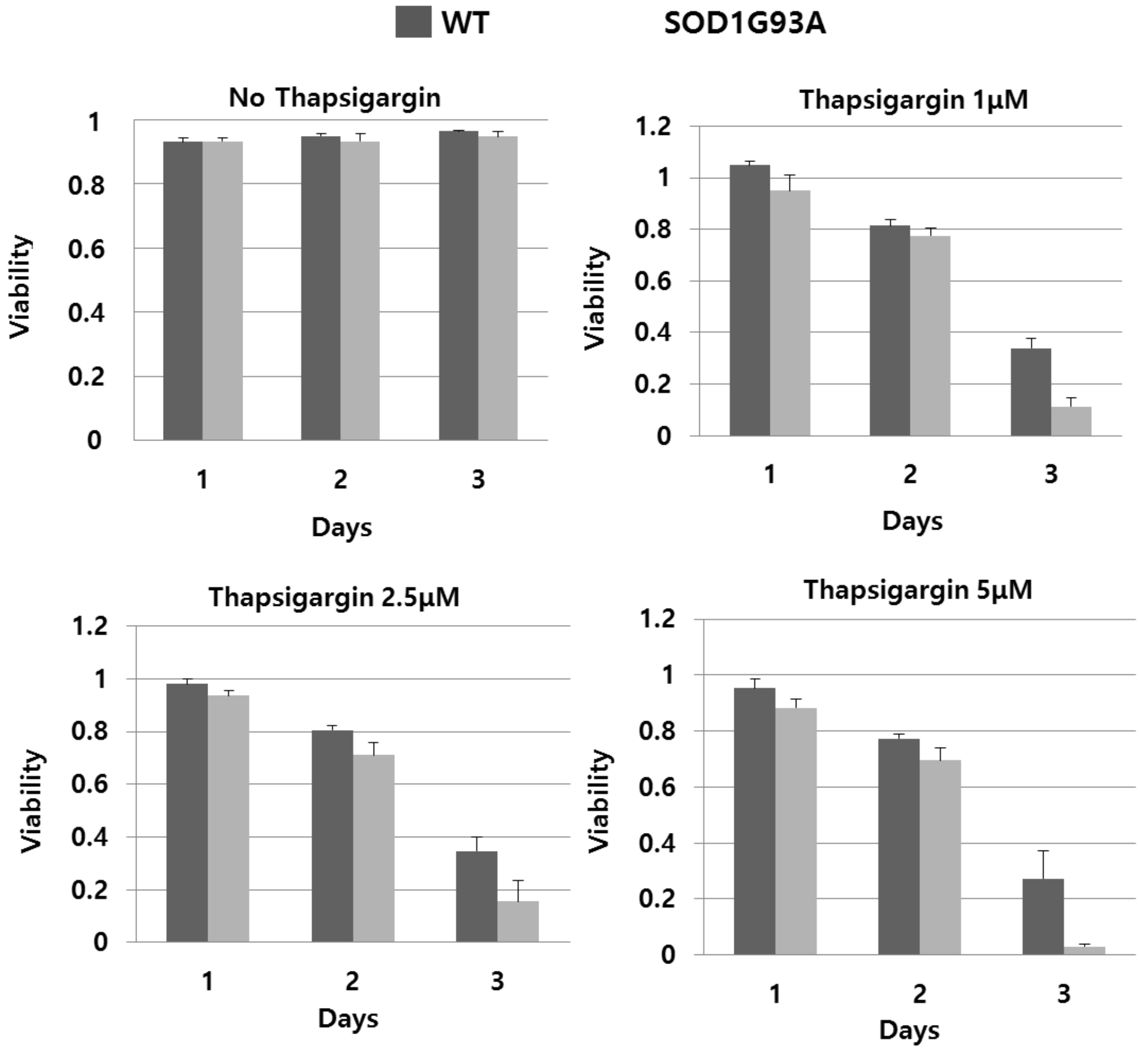

Figure S7. Enhanced cell death in SOD1G93A genotype neurons under ER stress. In the cell viability test with different concentrations of thapsigargin, SOD1G93A genotype neurons were much more susceptible to ER stress than WT.

a

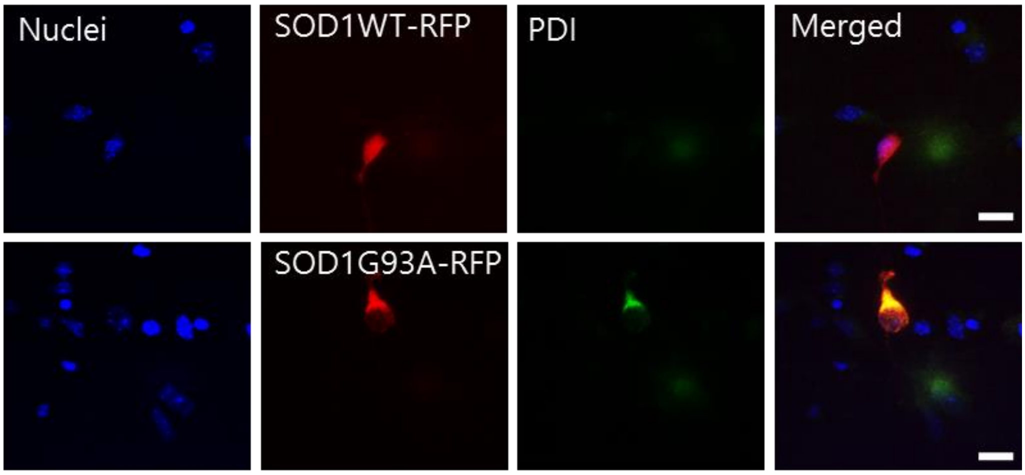

b

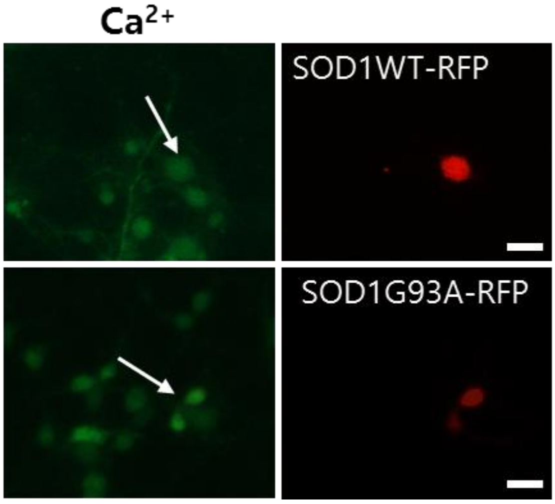

Figure S8. Increase the ER stress markers in SOD1G93A genotype neurons with treatment of 1 μM thapsigargin. The presence of SOD1G93A induced PDI expression (a) and Ca^{2+} release (b) under weak ER stress. (a) After treatment of 1 μM thapsigargin, PDI was upregulated in neurons expressing SOD1G93A-RFP at 1 μM thapsigargin for 12 h (down) but not neurons expressing SOD1WT-RFP (upper). (scale bar is 10 μm). (b) After treatment of 1 μM thapsigargin, Ca^{2+} was released in SOD1G93A expressing neurons (down, white arrow) but not in SOD1WT-RFP expressing neurons (upper, white arrow). (scale bar is 10 μm).

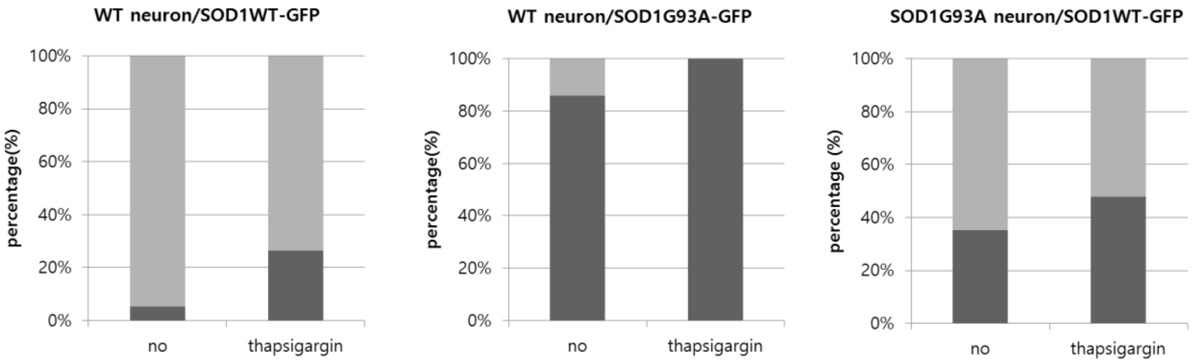

Figure S9. Inhibition of nucleic translocation of SOD1 protein under ER stress. After treatment of 1 μM thapsigargin for one day, the nucleic transportation of SOD1 protein was quantified. The number of neurons in which SOD1 protein translocated into the nucleus was reduced by the induced ER stress with 1 μM thapsigargin**.**

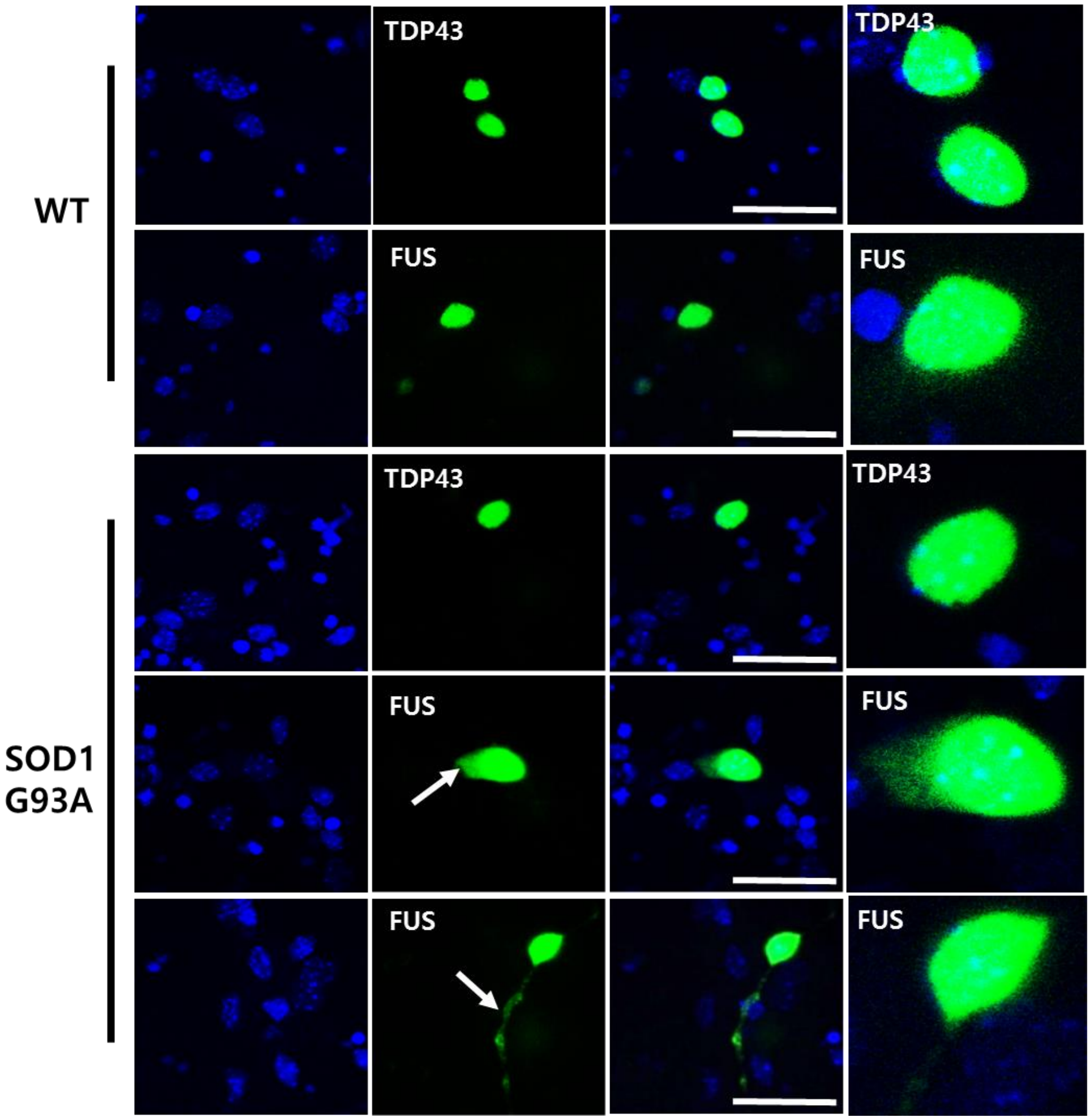

Figure S10. Localization of TDP-43 and FUS proteins in SOD1WT and SOD1G93A genotype neurons. The nucleic localization of TDP-43 was not interfered with in the presence of SOD1G93A, whereas some FUS protein was mislocalized in the cytoplasm in the SOD1G93A genotype neurons. (scale bar is 50 μm).

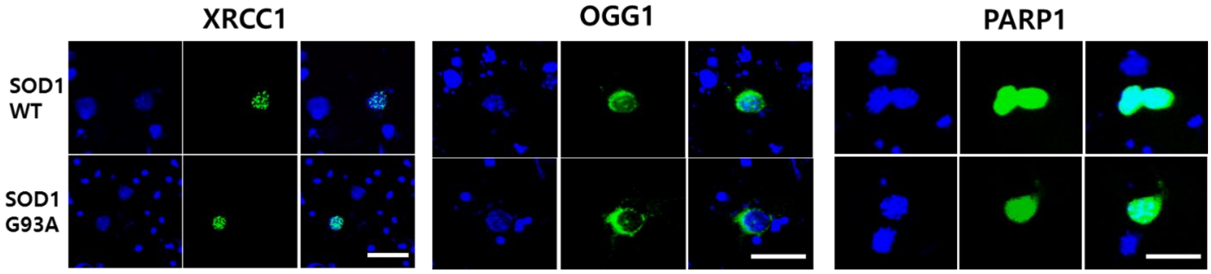

Figure S11. Nucleic localization of XRCC and OGG1. The presence of SOD1G93A did not affect the cellular localization of GFP-tagged XRCC1, OGG1, and PARP1. (scale bar is 20 μm).

a

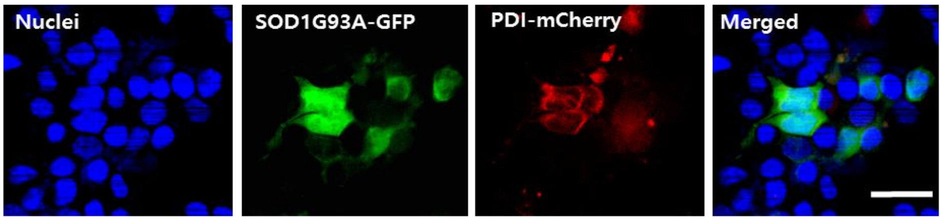

b

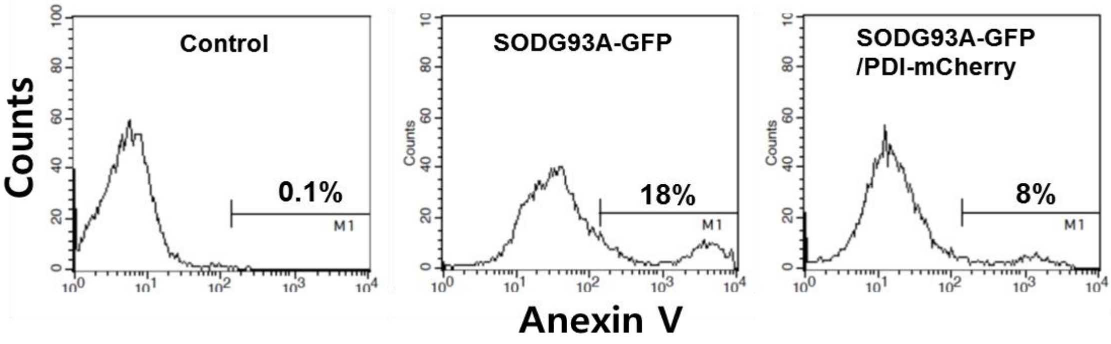

Figure S12. Transport of SOD1G93A into nuclei and rescue of cellular apoptosis by PDI overexpression in NSC34 cells. (a) Import of SOD1G93A-GFP (green) into nuclei by overexpressed PDI-mCherry (red). Cytoplasmic localized SOD1G93A-GFP in NSC34 cells was imported into the nuclei by overexpressed PDI-mCherry. (scale bar is 20 μm) (b) Rescue of cell death in cells expressing SOD1G93A by the overexpressed PDI-mCherry. Apoptosis of cells expressing SOD1G93A-GFP under ER stress was recovered by the overexpressed PDI-mCherry (right). (n=10000 neurons for control, SOD1G93A-GFP expressing and SOD1G93A-GFP/PDI-mCherry expressing neurons).
